# Insights into the Pathogenesis of Anaplastic Large-Cell Lymphoma through Genome-wide DNA Methylation Profiling

**DOI:** 10.1016/j.celrep.2016.09.018

**Published:** 2016-10-04

**Authors:** Melanie R. Hassler, Walter Pulverer, Ranjani Lakshminarasimhan, Elisa Redl, Julia Hacker, Gavin D. Garland, Olaf Merkel, Ana-Iris Schiefer, Ingrid Simonitsch-Klupp, Lukas Kenner, Daniel J. Weisenberger, Andreas Weinhaeusel, Suzanne D. Turner, Gerda Egger

**Affiliations:** 1 Clinical Institute of Pathology, Medical University of Vienna, 1090 Vienna, Austria; 2 Health & Environment Department, Molecular Diagnostics, Austrian Institute of Technology (AIT), 1190 Vienna, Austria; 3 Department of Urology, Norris Comprehensive Cancer Center, University of Southern California-Los Angeles, Los Angeles, CA 90089, USA; 4 Division of Molecular Histopathology, Department of Pathology, University of Cambridge, Cambridge CB2 0QQ, UK; 5 Ludwig Boltzmann Institute for Cancer Research, 1090 Vienna, Austria; 6 Unit of Pathology of Laboratory Animals (UPLA), University of Veterinary Medicine Vienna, 1210 Vienna, Austria; 7 Department of Biochemistry and Molecular Biology, Norris Comprehensive Cancer Center, University of Southern California-Los Angeles, Los Angeles, CA 90089, USA; 8 European Research Initiative on ALK-Related Malignancies (ERIA), Cambridge CB2 0QQ, UK

## Abstract

Aberrant DNA methylation patterns in malignant cells allow insight into tumor evolution and development and can be used for disease classification. Here, we describe the genome-wide DNA methylation signatures of NPM-ALK-positive (ALK+) and NPM-ALK-negative (ALK−) anaplastic large-cell lymphoma (ALCL). We find that ALK+ and ALK− ALCL share common DNA methylation changes for genes involved in T cell differentiation and immune response, including *TCR* and *CTLA-4*, without an ALK-specific impact on tumor DNA methylation in gene promoters. Furthermore, we uncover a close relationship between global ALCL DNA methylation patterns and those in distinct thymic developmental stages and observe tumor-specific DNA hypomethylation in regulatory regions that are enriched for conserved transcription factor binding motifs such as AP1. Our results indicate similarity between ALCL tumor cells and thymic T cell subsets and a direct relationship between ALCL oncogenic signaling and DNA methylation through transcription factor induction and occupancy.

## Introduction

Anaplastic large-cell lymphoma (ALCL) is a category of non-Hodgkin lymphoma (NHL) and is of T cell origin. ALCL is mostly diagnosed in children and young adults and represents 10%–15% of pediatric and adolescent NHL (Greer et al., 1991; Stein et al., 1985). A specific translocation (t(2;5)(p23q35)) involving fusion of the ubiquitously expressed nucleolar shuttling protein Nucleophosmin (NPM1) to the receptor tyrosine kinase anaplastic lymphoma kinase (ALK) is detected in more than 30% of ALCL cases (Morris et al., 1994). This translocation leads to the expression of the constitutively active NPM-ALK kinase, which is a potent trigger of multiple signaling pathways and is sufficient to drive cells toward malignant transformation (Bischof et al., 1997; Chiarle et al., 2003; Fujimoto et al., 1996; Turner et al., 2003). In NPM-ALK-positive (ALK+) ALCL, it has been shown that most changes leading to cell transformation are induced by transcription factors (TFs) such as STAT3/5, CEBPB, and AP1 (Leventaki et al., 2007; Mathas et al., 2002; Piva et al., 2006; Rassidakis et al., 2005; Turner et al., 2007; Zamo et al., 2002). In NPM-ALK-negative (ALK−) ALCL, recurrent driver mutations in *JAK1* and *STAT3* genes, as well as chimeras combining TFs with tyrosine kinases (*ROS1* or *TYK2*) and overexpression of truncated *ERBB4* transcripts, have been described (Crescenzo et al., 2015; Scarfò et al., 2016). ALK− ALCL shows a more heterogeneous genome in terms of somatic mutations and fusion transcripts, and gene expression studies indicate that ALK+ ALCL and ALK− ALCL display similar signatures, thus suggesting a common cell of origin (Boi et al., 2015; Eckerle et al., 2009).

Epigenetic alterations in cancer cells typically comprise global loss and local gain of DNA methylation (Egger et al., 2004). The latter has its largest impact on gene expression when found at promoter sites, because methylation at these sites is associated with silencing of the underlying genes. Changes in the methylome of malignant tissue contribute to dysfunctional gene expression and regulation. In addition, it has been shown that DNA methylation fingerprints of cancer tissues share distinct methylated sequences with their tissues of origin that make it possible to identify the stage of differentiation most closely related to the tumors and enable prediction of the cell of origin by epigenetic memory, which can be more reliable than by gene expression (Fernandez et al., 2012).

In ALK+ ALCL, only a few aberrantly methylated genes, including components of the T cell receptor (TCR) pathway and genes important for cell proliferation and survival, such as *p16^INK4A^*, *TNF-α*, *NFATC1*, *IL-2R*, and *BIM*, have been identified; even less is known about specific methylation changes in ALK− ALCL (Akimzhanov et al., 2008; Ambrogio et al., 2009; Martin-Subero et al., 2009; Nagasawa et al., 2006; Piazza et al., 2013; Zhang et al., 2009, 2011). Mechanistically, the TF STAT3 has been implicated in epigenetic silencing by upregulating and recruiting DNMT1 to promoters of some of the silenced genes in ALK+ ALCL (Zhang et al., 2005, 2007). However, no comprehensive genome-wide analysis of DNA methylation in ALCL has been conducted. In addition, it is unknown whether the presence of the ALK oncogene has an impact on DNA methylation in ALK+ compared to ALK− lymphomas.

Controversy also exists about the cell of origin of ALCL. Historically, mature T cells were presumed to be the targets for transformation, based on gene expression profiling and the presence of surface markers such as CD4 or CD30, as well as production of cytotoxic proteins such as Granzyme B and Perforin (Benharroch et al., 1998; Eckerle et al., 2009; Swerdlow et al., 2008). However, ALK+ cells are present in stem cell-enriched cord blood, and a study has identified a side population of cells in ALK+ ALCL cell lines and tumors with signatures similar to early thymic progenitors (ETPs), suggesting that transformation of T cells by NPM-ALK might occur early during thymic T cell development (Laurent et al., 2012; Moti et al., 2015). Subsequently, a mechanism has been proposed whereby in a murine mimic of ALK+ ALCL transient expression of a functional TCR is required for thymic emigration of incipient, thymic-resident tumor cells, but the TCR is then downregulated for peripheral lymphomagenesis (Malcolm et al., 2016).

We reasoned that a comprehensive study of the ALCL methylome might help to elucidate the impact of ALK-specific signaling on the epigenome of ALK+ ALCL in relation to ALK− ALCL and allow for a better classification of ALCL tumor cells with regard to their cellular origin.

## Results

### Identification of Methylation Variable Positions between Primary ALCL and Healthy Activated CD3^+^ T Cells

We performed DNA methylation profiling of human ALK+ (n = 5) and ALK− (n = 5) tumors and peripheral blood activated CD3^+^ T cells from healthy donors (n = 5) using the Illumina Infinium HumanMethylation450 BeadChip, which covers 482,421 CpG dinucleotides and 99% of RefSeq genes. An overview of the data analysis process is depicted in Figure S1.

**Figure 1:**
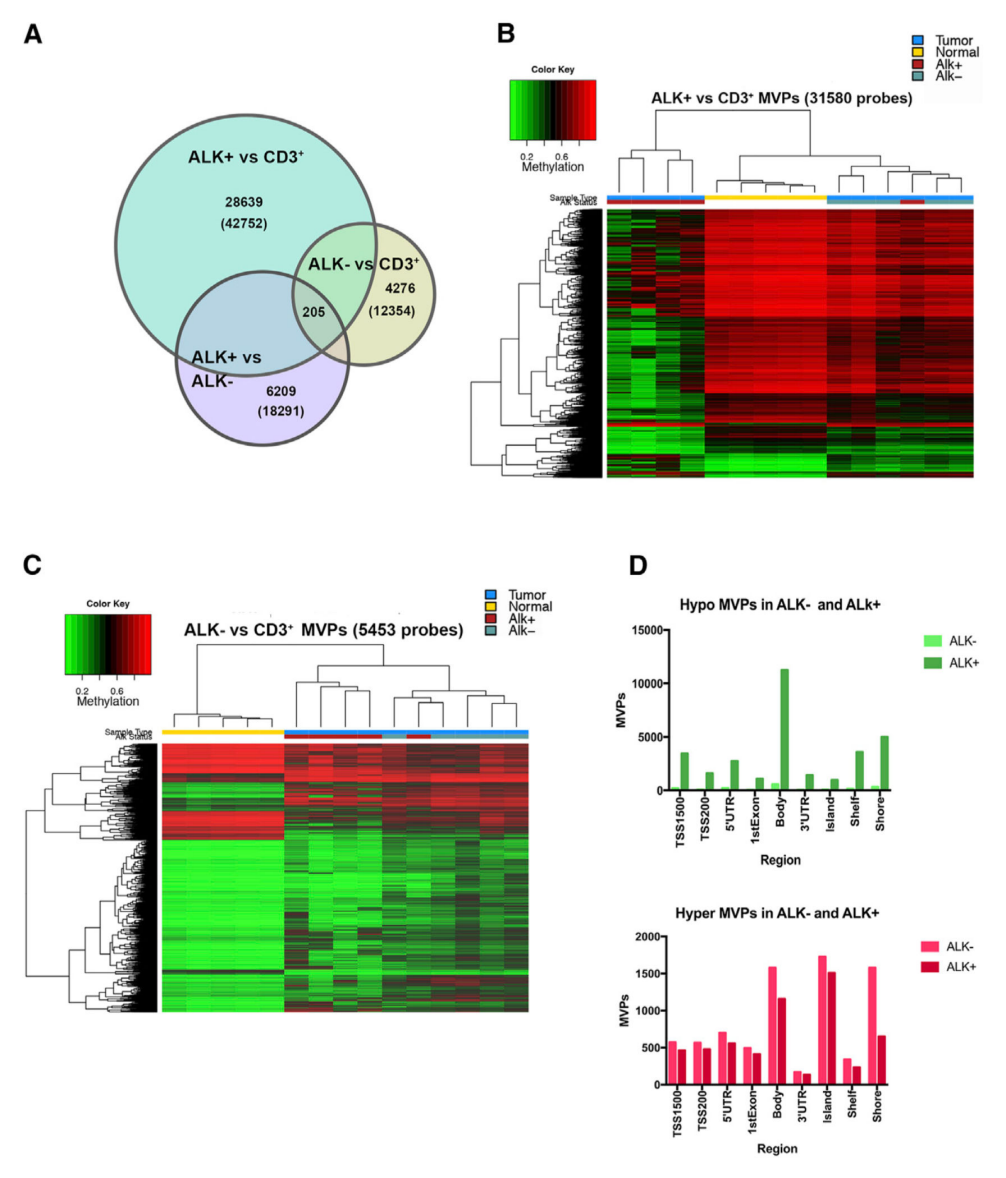
DNA Methylation of ALCL versus Normal CD3^+^ T Cells (A) Venn diagram showing significant MVPs (based on M values and adjusted p < 0.01) among indicated groups in comparisons of ALK+, ALK−, and normal CD3^+^. Numbers indicate unique MVPs of selected segments; numbers in parentheses indicate total number of MVPs of the three group comparisons: ALK+ versus CD3^+^, ALK− versus CD3^+^, and ALK+ versus ALK−. (B) Hierarchical clustering of the top 31,580 differentially methylated CpG sites detected between ALK+ and control T cells (color key indicates percentage of methylation, from red = 100% methylation to green = 0% methylation). Sample annotation: blue, tumor; yellow, normal CD3^+^; red, ALK+ ALCL; green, ALK− ALCL. (C) Hierarchical clustering of the top 5,453 MVPs detected in control T cells versus ALK− ALCL (color key and sample annotation as in B). (D) Number and distribution of MVPs between CD3^+^ T cells and ALK+ or ALK− ALCL found at specific genomic regions and regions around CpG islands. Hypomethylated MVPs are shown at the top; hypermethylated MVPs are at the bottom. Differentially methylated sites were obtained after filtering the data (filtering criteria: adjusted p value < 0.01 and β-value difference > |0.2|). See also Figures S1 and S2.

After data normalization and quality control steps, we identified 42,752 and 12,354 significant methylation variable positions (MVPs) between tumor (ALK+ or ALK−) and normal control (CD3^+^ T cells) (based on M value calculation and adjusted p < 0.01) (Figure 1A). Comparison of ALK+ to ALK− tumors revealed 18,291 significant MVPs. To perform hierarchical clustering and investigate the relationship among ALK+, ALK−, and CD3^+^ samples, we next filtered these MVPs with filtering criteria of an adjusted p < 0.01 and β-value difference > 0.2. Hierarchical clustering of ALK+ versus CD3^+^ cells (31,580 CpG sites) resulted in a distinct ALK+ cluster (except for one tumor) that appeared more distant to ALK− and CD3^+^ T cell samples because of a higher number of hypomethylated MVPs in ALK+ tumors (Figure 1B). However, MVPs differentiating between ALK− and T cells (5,453 probes) showed similar methylation in ALK+ ALCL (Figure 1C). In total, we identified 2,488 common MVPs in ALK+ and ALK− tumors, compared to peripheral T cells from healthy donors (Figure S2A). The major fraction of MVPs with differential methylation between ALK+ and ALK− tumors (8,147 sites) was hypomethylated in ALK+ (Figure S2B) and mainly located in gene bodies (Figure 1D, top panel). The frequency and localization of hypermethylated MVPs were highly similar in ALK+ and ALK− tumors (Figure 1D, bottom panel). Overall, these data demonstrate that many MVPs are shared between the tumor samples in comparison to normal CD3^+^ T cells and unique DNA methylation characteristics between ALK+ ALCL and ALK− ALCL are mainly based on hypomethylated MVPs in ALK+ tumors.

### DNA Methylation Patterns of ALCL Are Similar to Thymic Progenitor Cells

**Figure 2:**
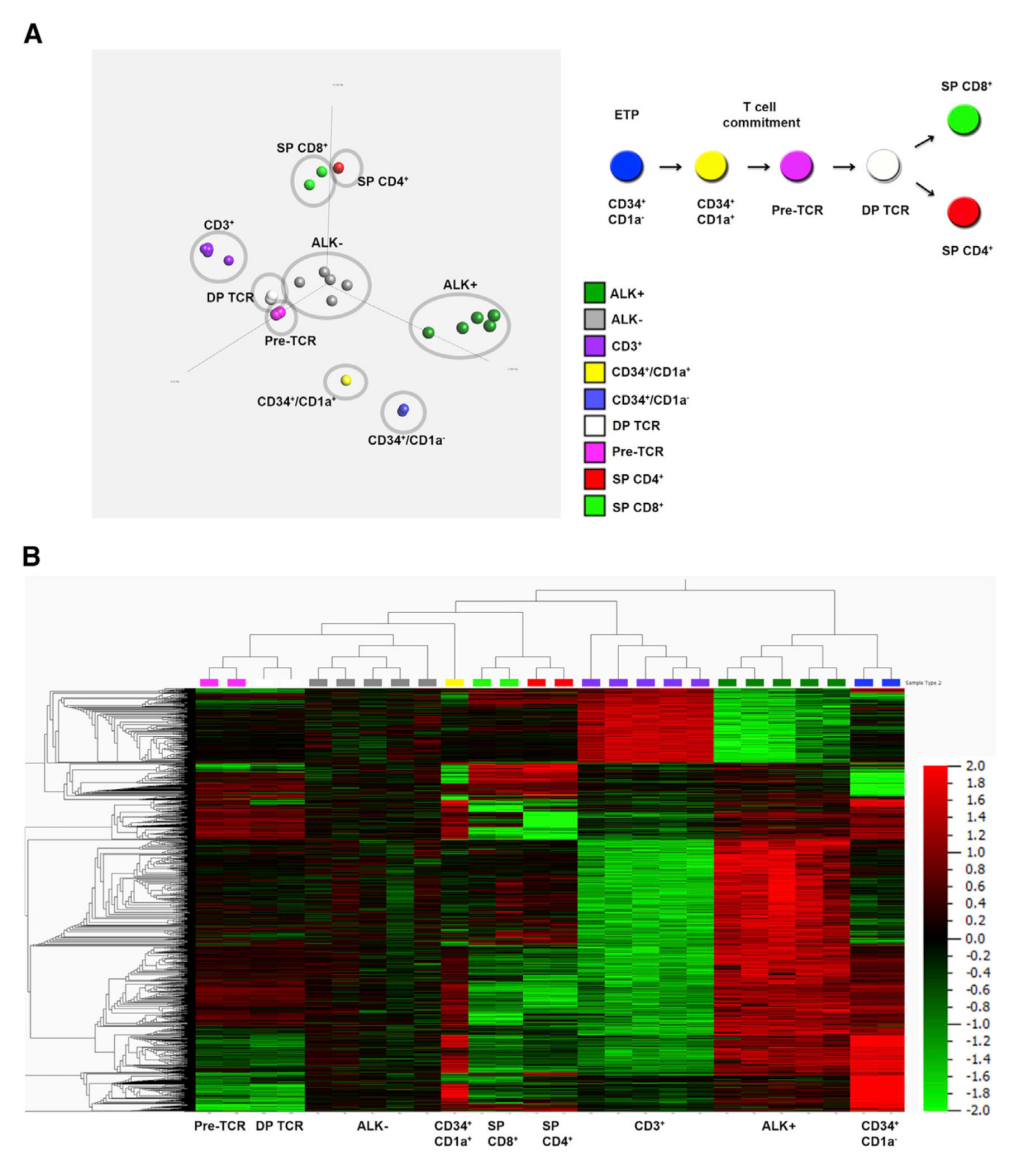
Comparison of Different Developmental Stages of Thymocytes with ALCL Tumor Cells (A) Left panel: principal-component analysis of thymic T cell subsets in comparison to ALK+ and ALK− tumor cells and peripheral CD3^+^ T cells (p < 9.4e–6, q value = 9.46e–4). Right panel: thymic developmental stages from ETPs (CD34^+^/CD1a^−^) to SP CD4^+^ or CD8^+^ cells. (B) Hierarchical clustering of the top 1% of all probes of thymic subsets, ALK+ and ALK− tumor cells, and peripheral CD3^+^ T cells (4,817 CpG sites) (p < 9.4e–6, q value = 9.46e–4). Data were normalized using Qlucore software, as described in the Supplemental Experimental Procedures. Global normalization was used to center the β values for each sample to a mean of 0 (variance = 1) to adjust for differences in signal intensities of the different Infinium BeadChips. Color key from green = −2 (0% methylation) to red = +2 (100% methylation).

**Figure 3:**
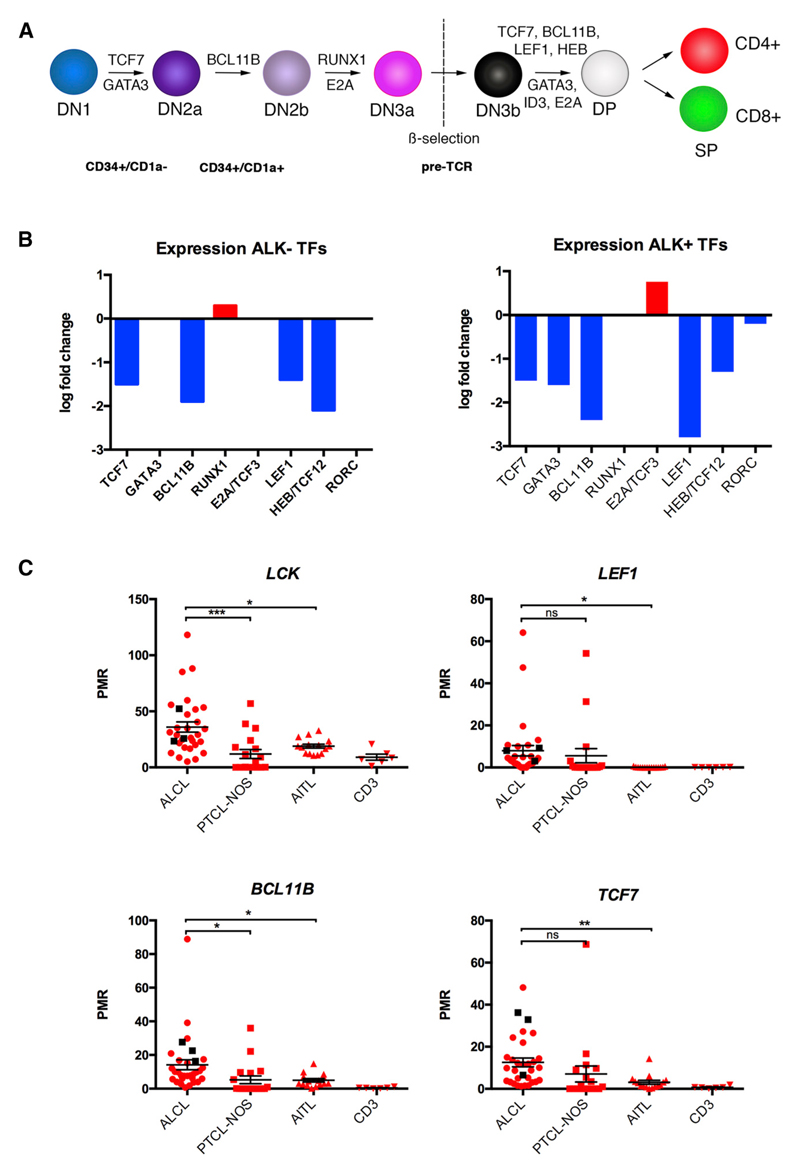
Silencing of T-Cell-Specific TFs in ALCL (A) Serial stages of thymic T cell development are driven by specific TFs. DN, double negative. (B) Gene expression differences of indicated TFs between ALK+ and ALK− ALCL compared to CD3^+^ T cells. (C) DNA methylation levels of promoter regions of indicated genes as determined by quantitative methylation ms-qPCR in 28 ALK+ ALCL, 3 ALK− ALCL, 15 AITL, and 18 PTCL-NOS tumor samples, with 6 healthy CD3^+^ samples as controls. Samples were analyzed by one-way ANOVA (p < 0.05) followed by pairwise comparisons to the control group using unpaired t tests. Values are shown as mean ± SEM. See also Figure S3.

Thymic development involves a complex process of differentiation from progenitor thymocytes to mature T cells and is associated with dynamic changes in DNA methylation in both mouse and human organisms. TFs and genes related to TCR signaling are methylated in progenitor cells and subsequently demethylated at different stages of T cell development (Ji et al., 2010; Rodriguez et al., 2015). To compare the ALCL methylome to different stages of T cell development, we integrated our data with publicly available methylation data of different developmental T cell stages (Rodriguez et al., 2015) using principal-component analysis and hierarchical clustering (p value < 9.4e–6). Comparison of the primary ALK+ and ALK− ALCL methylomes with multipotent ETP (CD34^+^/CD1a^−^), T cell committed progenitors (CD34^+^/CD1a^+^), pre-TCR T cells, TCR-expressing T (CD4^+^/CD8^+^ double positive [DP]) cells, and single positive (SP) CD4^+^ or CD8^+^ cells revealed that the DNA methylation pattern of ALK+ tumor cells resembled that of ETP (CD34^+^/CD1a^−^), whereas the ALK− methylome more closely resembled pre-TCR and DP TCR-expressing T cells (Figure 2A). These data were also reflected in hierarchical clustering analysis using the top 1% MVPs (4,817 CpGs, p < 9.4e–6, q value = 9.46e–4), indicating a close relationship among ALCL tumor cells, T cell progenitors, and early T cell stages (Figure 2B). These 4,817 MVPs were located in 2,526 individual genes, 10% of which were associated with transcription regulator activity according to gene ontology (GO) analysis (data not shown). This prompted us to analyze DNA methylation and expression levels of several T-cell-specific TFs that are essential for defined stages of thymic T cell development from ETP to SP T cells (Figure 3A). We detected high levels of DNA methylation in the promoters of *TCF7*, *GATA3*, *BCL11B*, and *LEF1* in ALK+ and ALK− tumors compared to CD3^+^ T cells from our dataset (Figure S3), which correlated with their decreased expression levels in tumors compared to CD3^+^ T cells, as identified by in silico analysis of previously published ALCL gene expression data (Figure 3B) (Eckerle et al., 2009). *GATA3* displayed lower promoter DNA methylation levels in ALK− ALCL, but no significantly different *GATA3* expression compared to CD3^+^ T cells was observed, which is consistent with the closer relationship of ALK− ALCL with DP TCR-positive cells based on DNA methylation analyses.

To corroborate these findings, we analyzed promoter DNA methylation of these TFs, as well as *LCK* promoter DNA methylation using methylation-sensitive qPCR (ms-qPCR) in a larger cohort (28 ALK+ and 3 ALK−) of ALCL patient samples (Figure 3C). We also compared these data to DNA methylation data of the two of the other most common peripheral T cell lymphoma subgroups, angioimmunoblastic T cell lymphoma (AITL, 15 samples) and peripheral T cell lymphoma, not otherwise specified (PTCL-NOS, 18 samples), and to normal CD3^+^ T cells. DNA methylation levels of both the *LCK* and the *BCL11B* promoters were significantly higher in ALCL compared to PTCL-NOS and AITL. *LEF1* and *TCF7* promoters were significantly hypermethylated in ALCL tumors compared to AITLs and to normal T cells, but no significant differences in DNA methylation levels were observed between ALCL and PTCL-NOS samples, most likely due to heterogeneity in the PTCL-NOS DNA methylation levels that is reflective of the diversity of this “wastebasket” disease category.

### Tumor-Cell DNA Methylation Reprogramming Is Associated with Epigenetic Modifications at Distinct Genomic Regions

**Figure 4:**
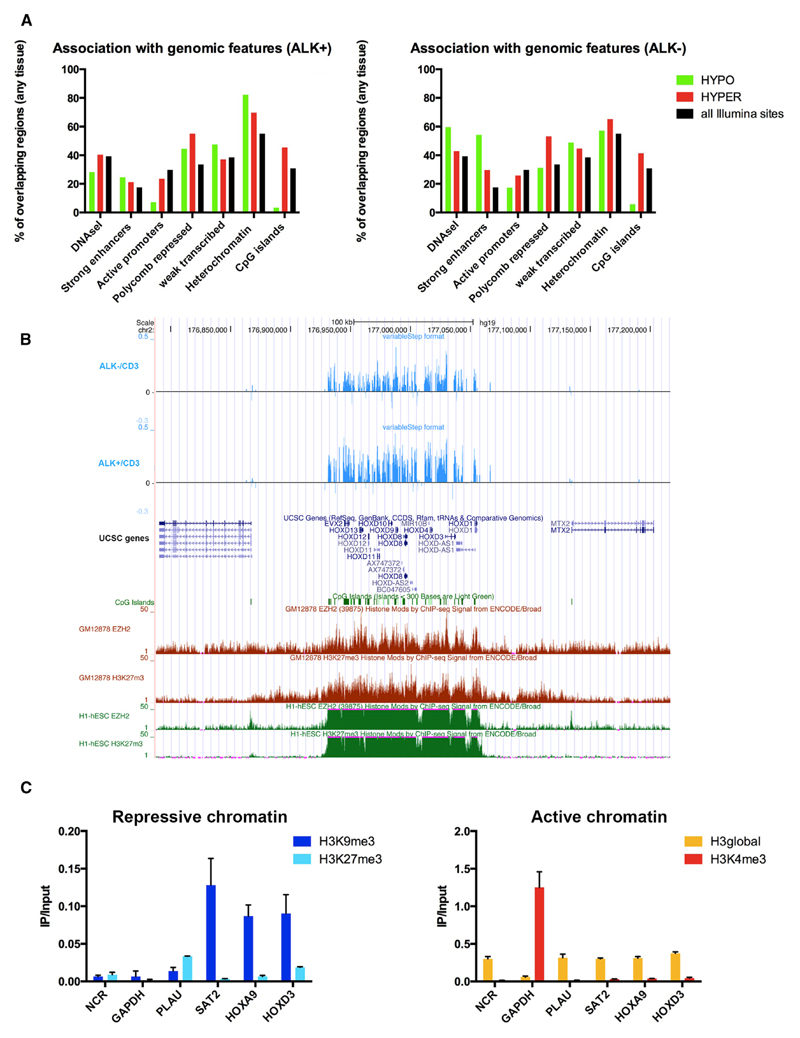
Genomic and Epigenomic Features of Differentially Methylated CpG Sites (A) Epiexplorer analysis using indicated genomic features of hypermethylated (red) and hypomethylated (green) CpG sites for ALK+ and ALK− ALCL compared to CD3^+^ T cells in relation to all CpG sites on the Illumina 450k array (black). (B) Epigenetic switching is detected at the *HOXD* cluster in ALK+ and ALK− ALCL at regions that show H3K27me3 and EZH2 occupancy by ChIP-seq in ESCs and in GM12878 lymphoblastoid cells. Top tracks (blue): differential methylation of ALK+/ALK− ALCL versus CD3^+^ T cells. Middle tracks: University of California Santa Cruz (UCSC) gene annotation track, where green boxes are CpG islands. Lower tracks: enrichment of EZH2 and H3K27me3 in lymphoblastoid cells (red) and ESCs (green). (C) ChIP interrogating repressive histone marks (H3K9me3, H3K27me3) and active histone marks (H3K4me3) at *HOXA9* and *HOXD3* gene promoters in ALK+ SU-DHL-1 cells. *GAPDH* is shown as control for an active region, *SAT2* is control for a heterochromatic region, NCR is control for a negative control region, and *PLAU* is control positive control for H3K27me3 occupancy. H3global indicates a control ChIP for global H3 occupancy. Values are means ± SD. Each value is the mean of three replicates. See also Figure S4.

We used the Epiexplorer web-based tool to associate regions of epigenetic reprogramming events in ALCL tumors with publicly available data (Halachev et al., 2012). As a control set, we selected the Illumina CpG annotation and compared our data to signatures from all tissues available in the Epiexplorer (Figure 4A). Hypomethylated MVPs in the ALK+ and ALK− versus CD3^+^ dataset were underrepresented at active promoters (7% and 17% versus 30%) and CpG islands (3% and 6% versus 31%), whereas hypermethylated MVPs showed enrichment at Polycomb-repressed sites (55% and 53% versus 33%) and CpG islands (45% and 41% versus 31%) compared to the control set. This is in line with previous findings showing that cancer-specific DNA hypermethylation is, besides CpG island methylation, also targeted to already silenced regions or regions harboring repressive histone marks, such as Polycomb-repressed genes (Shen and Laird, 2013).

The change from silencing by Polycomb repression in stem cells to DNA methylation in cancer cells is termed “epigenetic switching” and has been observed in different cancers (Gal-Yam et al., 2008; Ohm et al., 2007; Schlesinger et al., 2007; Teschendorff et al., 2010; Widschwendter et al., 2007). To test for the presence of epigenetic switching, we explored DNA methylation of the *HOX* gene clusters and other selected Polycomb group (PcG) target genes in embryonic stem cells (ESCs). The four *HOX* gene clusters (A–D) are known PcG targets in ESCs. As exemplified by the *HOXD* cluster, we detected DNA hypermethylation at all four clusters and additional homeobox genes in ALK+ and ALK− ALCL tumor samples (Figure 4B; Figure S4). Comparative analysis of these ALCL hypermethylated CpG sites with ENCODE chromatin immunoprecipitation sequencing (ChIP-seq) data showed that PcG occupancy, as defined by histone H3 lysine trimethylation (H3K27me3) and EZH2 binding, are present in ESCs and GM12878 lymphoblastoid cells at these sites (Bernstein et al., 2012; ENCODE Project Consortium, 2012). In contrast to the situation in ESCs and lymphoblastoid cells, but in line with epigenetic switching, we detected more than 10-fold enrichment of histone H3K9me3, a repressive chromatin mark that is frequently found in heterochromatic regions and regions with high DNA methylation, at *HOXA9* and *HOXD3* promoters and a relative depletion of PcG-related H3K27me3 in the ALK+ cell line SU-DHL-1 (Figure 4C). H3K4me3, which is associated with active promoters, was also depleted at these loci.

Altogether, these data show a significant difference in genome-wide DNA methylation between ALCL tumors and normal activated T cells in regions that control transcriptional activity of genes, CpG islands and PcG-targeted regions in ESCs, which appears to be a common feature of epigenetic tumor cell reprogramming.

### Identification of Differentially Methylated Regions between Tumor and Normal T Cells and Their Relation to Altered Gene Expression

**Figure 5:**
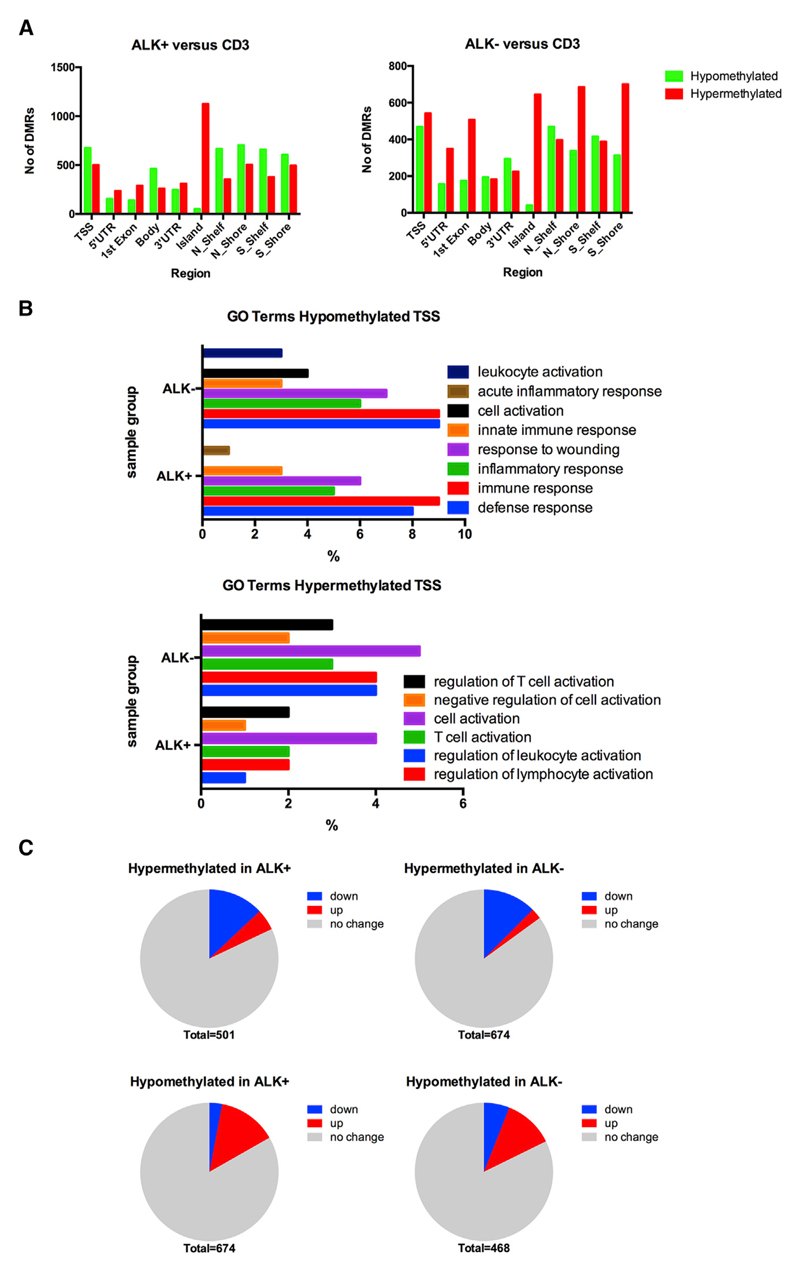
Characterization of DMRs (A) Identification of DMRs, calculated from mean β values of all CpGs annotated to a distinct genomic region, between ALK+ (left) and ALK− (right) versus CD3+ T cells (p value < 0.05; β-value difference ≥ 0.15). TSS includes CpGs within either 200 or 1,500 bp of the TSS. Hypermethylated DMRs are depicted in red; hypomethylated DMRs are in green. (B) GO term analysis using the DAVID web-based tool. Significant GO terms (adjusted p < 0.05) are highly similar in ALK+ and ALK− tumors. (C) Correlation of genes with differentially methylated promoters with gene expression profiles of ALK+ and ALK− ALCL and T cells. Blue, genes downregulated in ALCL versus T cells; red, genes upregulated in ALCL versus T cells. Hypermethylated TSS, genes showing higher methylation in both groups within their promoters; hypomethylated TSS, genes with lower methylation in their promoters in both groups. See also Tables S1, S2, and S3.

Next, we sought to identify distinct genomic regions spanning multiple CpG sites that showed differential methylation between tumor and healthy control samples. To identify such differentially methylated regions (DMRs) between samples, mean DNA methylation levels of multiple probes were calculated for distinct regions based on the annotated Illumina categories. These categories included gene promoters spanning regions 1,500 and 200 bp upstream of transcription start sites (TSSs) (TSS1500 and TSS200), 5′ UTRs, first exons, gene bodies, 3′ UTRs, CpG islands, CpG island shores, and CpG island shelves (defined as regions surrounding CpG islands). Data were filtered according to criteria expected to reflect biologically significant differences in DNA methylation (adjusted p value < 0.05; β-value difference ≥ 0.15) (Figure 5A). Generally, the numbers of hypermethylated and hypomethylated DMRs in ALK+ and ALK− samples compared to controls were similar, except for more hypomethylated DMRs located in TSSs (674 ALK+, 468 ALK−), gene bodies (462 ALK+, 194 ALK−), and regions adjacent to CpG islands (e.g., shelves, and shores) (658 on average in ALK+, 384 on average ALK−) in ALK+ samples. ALK− samples in general showed larger numbers of hypermethylated DMRs, especially in the 5′ UTR and first exon region and in CpG island shores. Regarding CpG islands, hypermethylated DMRs were detected in both ALK+ and ALK− samples (1,126 ALK+, 645 ALK−), but only 52 and 40 CpG islands, respectively, were hypomethylated. This was expected, because CpG islands are usually kept free of methylation in normal somatic cells, independent of the expression level of their underlying gene (Jones et al., 1990). Most hypomethylated islands were located in non-promoter CpG islands in transcribed regions at the 3′ end of genes or in gene deserts. Almost no significant DMRs could be identified between ALK+ and ALK− samples, suggesting that no ALK-specific influence on promoter DNA methylation, and thus gene expression, can be detected (data not shown).

To identify biologically relevant functions and pathways affected by DNA methylation changes, we performed GO term analysis on differentially methylated gene promoters using the Database for Annotation, Visualization and Integrated Discovery (DAVID) (Huang et al., 2009a, 2009b). We interrogated the database for significant differentially methylated genes with hypermethylated or hypomethylated promoters, assuming that altered gene promoter DNA methylation is associated with changes in gene expression between healthy and tumor samples. Promoter DNA methylation in both ALK+ and ALK− tumors showed a large overlap of enriched GO terms. Hypomethylated promoters were implicated in biological processes such as immune or defense response (Figure 5B; Table S1). Hypermethylated promoters of both tumor groups were associated with high significance with GO terms related to immune system processes such as “regulation of immune cell activation,” specifically “T cell activation” (Figure 5B; Table S1).

To determine the relationship of DNA methylation changes and significant alterations in biological activity, we evaluated the correlation of promoter DMRs with changes in gene expression between CD3^+^ T cells and ALK+ and ALK− ALCL. To this end, we used publicly available gene expression data on ALK+ and ALK− ALCL and CD4/CD8 T cell subsets and correlated these to DMRs in promoters identified from our data (TSS200, TSS1500) (Figure 5C) (Eckerle et al., 2009). In ALK+ tumors, 65 of 501 (13%) significantly hypermethylated TSSs were found to be significantly downregulated, and 93 of 674 (14%) hypomethylated TSSs were upregulated by more than 1.5-fold. Similarly, in ALK− tumors, 84 of 674 (12%) hypermethylated and 55 of 468 (12%) hypomethylated DMRs were down- and upregulated by more than 1.5-fold, respectively. Top differentially methylated and deregulated genes are listed in Table S2. GO term analysis of this dataset of both differentially methylated and deregulated genes confirmed their association with immune cell-related processes such as T cell activation for hypermethylated silenced genes and defense response for hypomethylated and upregulated genes for both tumor subgroups (Table S3). In addition, we identified 25 and 17 hypermethylated gene promoters with increased gene expression, as well as 20 and 28 hypomethylated gene promoters with decreased expression, in ALK+ and ALK− samples, respectively. However, this discrepancy may be because all affected genes were represented by alternative transcripts, whereas the altered DNA methylation was only identified in one specific isoform that might not represent the isoform determined in the expression dataset.

Altogether, these data show that differential methylation of genes in ALCL affects mainly biological processes related to immune cell function, which reflects the characteristics of the transformed T cells, and is associated with altered gene expression in approximately 12% of hypo- and hypermethylated genes in both tumor subgroups.

### Major T-Cell-Specific Pathways and Networks Are Associated with Altered DNA Methylation

**Figure 6:**
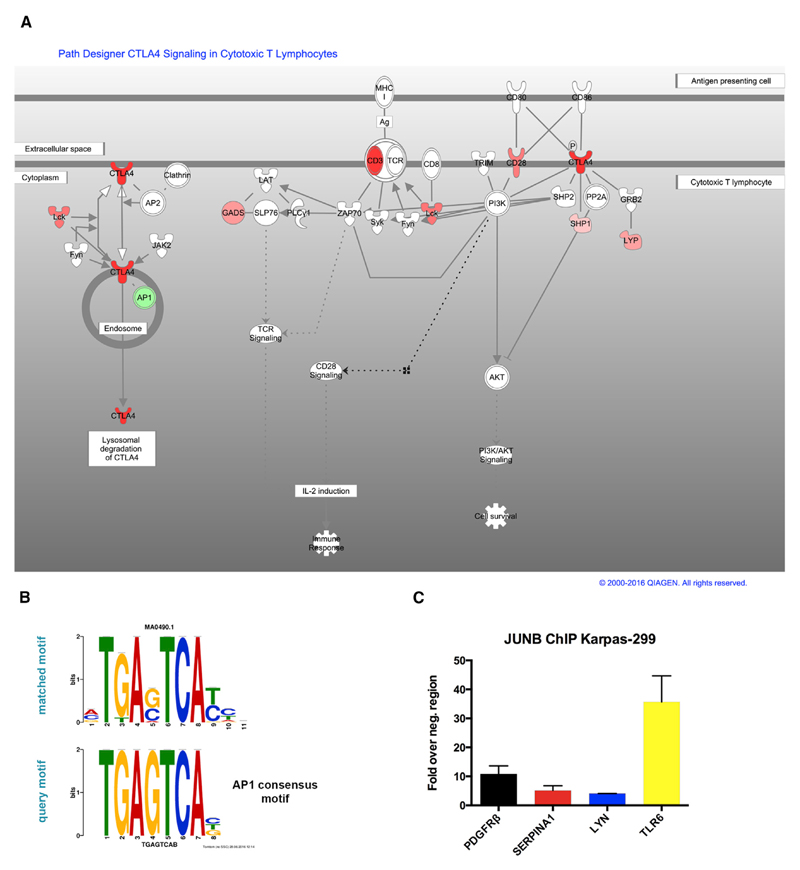
Canonical Pathways Are Affected by Differentially Methylated Genes (A) Multiple genes of the TCR pathway are hypermethylated in ALK+ and ALK− ALCL (red, significantly hypermethylated genes; green, significantly hypomethylated genes; adjusted p value < 0.05; β-value difference ≥ 0.15). The pathway was generated through the use of QIAGEN’s IPA. (B) DNA sequence motif identified by unbiased motif search in regions adjacent to hypomethylated CpGs in ALK+ and ALK− ALCL (top) compared to the AP1 consensus motif (bottom). (C) ChIP for JUNB occupancy at hypomethylated ALCL promoters with putative AP1 binding sites. *PDGFRβ*, positive control. ChIP was normalized to a negative control region in the 3′ end of the *PDGFRβ;* gene containing no AP1 motif. Values are means ± SEM. Each value is the mean of three replicates. See also Figures S5–S8.

To obtain a better understanding of cellular networks and pathways affected by altered DNA methylation in ALCL, we next performed Ingenuity Pathway Analysis (IPA) (http://www.ingenuity.com). Due to the high similarity of promoter DMRs in ALK+ and ALK− samples, we used the significant 194 hypomethylated and 186 hypermethylated overlapping promoters from both tumor subgroups. We identified the TCR and CTL4-A pathways as being among those canonical pathways most significantly affected by DNA hypermethylation (Figure 6A). Seven genes implicated in the TCR and CTLA-4 signaling pathways (*CD3*, *CD28*, *CTLA-4*, *LCK*, *GADS*, *SHP1*, and *LYP*) showed hypermethylation in ALCL samples. These data are in line with a previous report of DNA hypermethylation of several TCR pathway genes in ALK+ ALCL (Ambrogio et al., 2009) (Figure S5).

Among hypomethylated gene promoters, TREM1 signaling scored as the top canonical pathway (Figure S6). TREM1 is a triggering receptor promoting cytokine and chemokine production (Bouchon et al., 2000). This finding supports the reported distinct cytokine expression profiles for ALK+ and ALK− ALCL tumors (Al-Hashmi et al., 2001; Iqbal et al., 2010; Lamant et al., 2007).

Using the Regulator Effects feature in IPA, we identified regulators and functions strongly linked with T cell accumulation, tumor cell proliferation, immune cell function, and cell expansion. In summary, known ALCL driving factors, including CEBPB, nuclear factor κB (NF-κB), mitogen-activated protein kinase (MAPK), and STAT3, were suggested as upstream regulators targeting the affected hypo- and hypermethylated genes identified from our datasets (Figure S7).

### Enrichment of TF Binding Site Motifs in TF Binding Site Motifs

Because deregulated TFs play a key role in ALK-dependent transformation and may be involved in reshaping the ALCL epigenome, we next sought to evaluate whether changes in the ALCL epigenome are related to specific TFs in primary ALCL. To identify enrichment of TF binding sites in the vicinity of CpG sites that are hypomethylated in ALK+ and ALK− ALCL tumors, we searched for common DNA sequence motifs in windows of 100 bp surrounding significant MVPs (28,255 hypomethylated CpG sites in ALK+ ALCL tumors and 1,291 hypomethylated CpG sites in ALK− ALCL tumors) using the MEME-ChIP tool and related applications within the Multiple EM for Motif Elicitation (MEME) web-based suite (Bailey et al., 2009; Machanick and Bailey, 2011). The top motif with the highest frequency in hypomethylated sites of ALK+ and in ALK− ALCL was a motif of 8 bases (e values 5.91e–3 and 5.19e–2, respectively) (Figure 6B). This motif is similar to the consensus motif of the basic leucine zipper TFs such as JUN, JUNB, JUND, and FOS, which belong to the AP1 family of TFs. ChIP in ALK+ Karpas-299 cells revealed binding of the AP1 factor JUNB at distinct top hypomethylated promoters, including *SERPINA1*, *LYN*, and *TLR6*, with putative AP1 binding sites (Figure 6C). ALCLs are highly dependent on JUNB, as indicated by decreased cell population doublings after short hairpin RNA (shRNA)-mediated depletion of JUNB in Karpas-299 cells. This reduction of cell doubling was mainly due to cell-cycle arrest in the G0/G1 phase and increased apoptosis (Figure S8). Altogether, our data show that AP1 is a major downstream regulator of NPM-ALK ALCL signaling, which is reflected in lower DNA methylation levels of genes containing AP1 binding sites in ALCL.

## Discussion

In cancer cells, changes in the methylome comprise focal DNA hypermethylation, together with genome-wide DNA hypomethylation of genomic regions (Egger et al., 2004). These changes depend on general aspects of cancer development, the cell of origin, and individual characteristics obtained by the malignant cell during transformation. For ALK+ ALCL, which largely depends on the oncogenic driver NPM-ALK, and ALK− ALCL, for which no single defined oncogenic driver has been identified as yet, no systematic analysis of global DNA methylation has been performed so far. This prompted us to study DNA methylation differences between the two tumor groups and to investigate the association of oncogene signaling and epigenetic alterations between normal T cells and lymphoma.

Regarding the origin of ALK+ and ALK− ALCL, our data suggest that progenitor thymic T cell stages, not mature SP CD4^+^ or CD8^+^ cells, are the sources of ALCL, because both ALK+ and ALK− ALCL DNA methylation patterns were highly similar to discrete developmental stages of thymocytes. Both ALK+ and ALK− lymphomas are classified, together with AITL and PTCL-NOS, as peripheral T cell lymphomas, which have–in contrast to, e.g., lymphoblastic lymphoma–by definition a post-thymic origin. However, our findings, which are based on comparison of DNA methylation patterns among different T cell differentiation stages, indicate that T cell transformation in ALK+ ALCL may occur in progenitor cells rather than in mature T cells and are in line with two papers reporting that ALCLs have a primitive origin and arise in thymocytes before TCR β-rearrangement (Malcolm et al., 2016; Moti et al., 2015). We found ALK+ ALCL DNA methylation patterns to be in proximity to CD34^+^/CD1a^−^ patterns corresponding to the ETP stage and DNA methylation patterns of ALK− ALCL to be close to those of DP (CD4^+^ CD8^+^) or pre-TCR-positive T cells. The ETP stage still has the potential to differentiate into B cell, myeloid, natural killer (NK), and dendritic cell (DC) lineages, and the close relationship of ALK+ ALCL to this stage might explain why myeloid antigen expression is frequently observed in ALK+ ALCL, thereby confusing an ALCL diagnosis (Popnikolov et al., 2000). In line with this, we found the TREM1 signaling pathway to be affected by DNA hypomethylation. TREM1 is a triggering receptor expressed on myeloid cells with a function in stimulating neutrophil- and monocyte-mediated inflammatory responses by triggering the release of pro-inflammatory cytokines and chemokines (Arts et al., 2013; Ford and McVicar, 2009). Thus, TREM1 signaling might be a consequence of lacking T cell commitment at the precursor stage following transformation by ALK and other oncogenic events. The peak occurrence of ALK+ ALCL in children and young adolescents, when thymic T cell development is most prominent, would lend support to an early origin of ALK+ ALCL. However, we cannot exclude that the similarity of the ALCL epigenome to thymic T cell subsets is due to reprogramming of mature T cells by oncogenic signaling and a regression to a progenitor phenotype.

In addition, ALK+ ALCL and ALK− ALCL revealed unique promoter DNA methylation of T-cell-specific TFs, thereby clearly distinguishing them from PTCL-NOS and AITL. Thymic T cell development follows a tightly regulated sequential process involving different stages, from early T cell commitment, to TCR rearrangement and β-selection, through to positive selection of SP CD4^+^ and CD8^+^ T cells. As shown by a study, normal T cell differentiation and TCR function are accompanied by specific DNA methylation changes, and TF demethylation, which is irreversible in most cases, is observed during the developmental process (Rodriguez et al., 2015). In contrast to normal differentiation, and specific for ALCL when compared to AITL and PTCL-NOS, we identified DNA hypermethylation and repression of the T-cell-specific TFs *LEF1*, *TCF7*, *GATA3*, and *BCL11B* in our sample cohort. This may indicate that TF demethylation required during normal differentiation does not take place or is profoundly perturbed in ALCL. In healthy T cells, the TFs found to be methylated in ALCL normally drive T cell development, together with NOTCH1 signaling. LEF1 and TCF7 are essential for the induction of T-cell-specific genes, including the TCRα enhancer (Okamura et al., 1998). In addition, the TCF7 target *BCL11B*, which is expressed during the early DN2 stage of mouse thymic development, is needed for T cell commitment (Li et al., 2010). The TF GATA3, which is crucial not only for T cell survival and commitment in early stages but also for the transition to the DP state in later stages (Yui and Rothenberg, 2014), was not hypermethylated in ALK− ALCL, and there was no expression difference when compared to CD3^+^ T cells, corresponding to the DNA methylation phenotype of ALK− ALCL tumors that more closely resemble the DP stage of thymic development. This might indicate that ALK− ALCLs, which predominantly occur in older patients, are not originating in thymic T cells. Overall, DNA methylation analysis of distinct T-cell-specific TFs could be used for diagnostic purposes for T cell lymphoma classification.

The unique ALCL-specific promoter DNA methylation of these key T cell TFs, resembling that of early T cell stages, suggests that differentiation to T cells with an activated phenotype is likely not driven by T cell TFs in ALCL but rather might be induced by oncogenic signaling by ALK and other oncogenes at this specific T cell stage. The observation of major in-frame clonal TCRα rearrangements in the absence of TCRβ clonal rearrangements in human ALK+ patients’ samples demonstrates that abnormal T cell differentiation processes take place in the thymus at these stages (Malcolm et al., 2016). In addition, data in mice suggest that some form of the TCR seems to be a prerequisite for thymic egress and for the activated T cell phenotype of ALCL, but it has been shown that the TCR has to be subsequently downregulated for lymphomagenesis by epigenetic silencing, which is also supported by our data (Ambrogio et al., 2009; Malcolm et al., 2016). Besides the TCR, we find the T-cell-specific receptor *CTLA-4* to be hypermethylated and repressed in ALCL. CTLA-4 is a receptor normally expressed on activated T cells that inhibits T cell activation by reducing interleukin-2 (IL-2) production and interleukin-2 receptor (IL-2R) expression and causing cell-cycle arrest (Alegre et al., 2001). Its activation is associated with reduced activation of the MAPKs ERK and JNK and inhibition of the TFs NF-κB, NFAT, and AP1, which play a major role in ALK-dependent transformation of T cells, suggesting that CTLA-4 downregulation by DNA methylation contributes to intact ALK signaling. Its importance for regulation of T cell proliferation has also been demonstrated in murine knockout models, because *Ctla-4* deletion led to accumulation of T lymphocytes in peripheral lymphoid and solid organs, thus resembling a T cell lymphoproliferative disorder (Tivol et al., 1995; Waterhouse et al., 1995). Thus, if the cell of origin in ALK+ ALCL is an ETP, survival or bypass of subsequent selection processes in the thymus may be accomplished by oncogenic signaling processes induced by ALK without the aid and presence of T-cell-specific TFs. In addition, deregulation of the TCR and CTLA-4 signaling could be an obligatory consequence of ALK signaling to enable the transgression of the disease to the periphery. Thus, reactivation of the TCR pathway and CTLA-4 signaling using DNA methyltransferase inhibitors might provide a therapeutic option for ALCL patients.

Overall, our data show that ALK+ ALCL and ALK− ALCL harbor similar methylation patterns relevant for gene expression compared to CD3^+^ cells. In direct comparison to ALK− ALCL, we observed that a larger number of certain genomic regions such as gene bodies were hypomethylated in ALK+ tumors, but surprisingly, we could not identify an ALK+-specific DNA methylation signature at gene promoters in ALK+ samples. This implies that signaling through hyperactive ALK does not lead to a distinct biologically relevant epigenetic change in ALK+ ALCL compared to ALK− ALCL. The overall similar methylation changes may be explained by analogous oncogenic driving forces, such as specific TFs, that are activated or inactivated in both ALK+ ALCL and ALK− ALCL. For example, NPM-ALK has been shown to induce promoter DNA methylation of tumor suppressors via its downstream target STAT3, which is also a highly relevant TF in ALK− ALCL (Crescenzo et al., 2015). Another common major oncogene-induced transcription factor in both ALK+ ALCL and ALK− ALCL is AP1 (Laimer et al., 2012; Staber et al., 2007). In our data, we observed conserved AP1 TF binding sites enriched when situated close to hypomethylated CpG sites in ALCL tumors. Enrichment of AP1 at hypomethylated CpG sites and protection from DNA methylation by the AP1 family member c-Jun has been described in colon cancer and in BCR-ABL-induced lymphoid leukemia (Berman et al., 2011; Kollmann et al., 2011). In line with this, we detected binding of the AP1 family member JunB at hypomethylated CpG sites in ALK+ cells and identified TFs such as STAT3, NF-κB, CEBPB, and MAPK as upstream transcriptional regulators of genes with hypo- and hypermethylated promoters. These findings indicate that specific TFs are associated with alterations in DNA methylation in both ALK+ ALCL and ALK− ALCL.

Despite the distinct DNA methylation changes, especially in ALK+ ALCL, suggesting a thymic progenitor, it cannot be ruled out that mature transformed T cells turn on an epigenomic program that may lead to reprogramming of the DNA methylome to that of a thymic progenitor. We detected such epigenetic reprogramming at PcG regions, which contain the passive H3K27me3 mark in stem cells. PcG target genes are involved in developmental processes and are often set to a poised state in ESCs, but they can be silenced at later stages of differentiation depending on the presence of repressive PcG complexes and histone marks. DNA methylation at these bivalent chromatin domains has been observed in a multitude of cancers and has been discussed as an epigenetic stem cell signature of cancer, resembling reprogramming of the cancer cell epigenome to a more dedifferentiated state (Gal-Yam et al., 2008; Lehnertz et al., 2003; Ohm et al., 2007; Tachibana et al., 2008; Viré et al., 2006; Widschwendter et al., 2007). This gain of methylation at genes important for stemness and differentiation could also explain the increased methylation detected at T-cell-specific TFs in ALCL. Still, no hypermethylation at these sites is detected in PTCL-NOS and AITL, indicating that promoter DNA methylation gain at T-cell-specific TFs is specific for ALCL and not a universal feature of other peripheral T cell lymphomas.

Altogether, our study identified distinct DNA methylation changes in ALK+ and ALK− ALCL tumors that are reflective of a thymic origin of ALCL and include major TFs and pathways related to immune cell function and development. We did not observe direct evidence for NPM-ALK-specific epigenetic alterations in our data, although deregulation of TFs important during T cell differentiation and development is accomplished via DNA hypermethylation and ALCL-specific TFs are involved in epigenetic changes of tumor cells at sites of DNA hypo- and hypermethylation.

## Experimental Procedures

Information on cell lines, DNA preparation, and bioinformatic analyses can be found in the Supplemental Experimental Procedures.

### Human Patient Samples and Controls

Archived fresh frozen tissue from ALK+ and ALK− patients (four female and one male each, tumor content > 90% histopathologically verified), and blood samples for CD3^+^ T cell isolation from five healthy female patients as controls were obtained in an anonymized fashion from the Institute of Clinical Pathology (Medical University of Vienna) and the St. Anna Children’s Hospital. For clinicopathological characteristics, see Table S4. Archived formalin-fixed paraffin-embedded (FFPE) tumors from 28 ALK+ and 3 ALK− ALCL patients, 18 PTCL-NOS patients, and 15 AITL patients were also obtained in a blind and randomized fashion from the Institute of Clinical Pathology. The study protocol for the use of patient tissues was approved by the Institutional Ethics Committee of the Medical University of Vienna (1224/2012).

### Isolation of CD3^+^ Cells from Whole-Blood Samples

Peripheral blood mononuclear cells (PBMCs) were isolated by density centrifugation of whole-blood samples (Lymphoprep) and bound to CD3 microbeads (Miltenyi). Bound cells were loaded onto an equilibrated MACS LS separation column (Miltenyi) and washed, and CD3^+^ cells were eluted according to the supplier’s protocol.

### DNA Methylation Analysis by Illumina Infinium HumanMethylation450 BeadChips

Before hybridization onto arrays, 0.5 μg of each DNA sample (5 ALK+ frozen tumors, 5 ALK− frozen tumors, and 5 CD3^+^ T cells) were bisulfite converted with the EZ DNA Methylation Kit (Zymo Research) according to the manufacturer’s protocol and Illumina’s recommendations. Bisulfite conversion was carried out overnight in a thermocycler at 95°C for 30 s and then 50°C for 60 min (16 cycles), and bisulfite DNA was purified and eluted in 12 μL of sterile water. Then, 4 μL of bisulfite-converted DNA were subjected to genome-wide amplification, following an isothermal incubation step at 37°C for 20 hr. After genome-wide amplification, the DNA was enzymatically fragmented, purified, and precipitated. The precipitated DNA was resuspended in 42 μL RA1 buffer and denatured at 95°C for 20 min. The denatured DNA samples (12 μL) were applied to the BeadChip for hybridization at 48°C for 20 hr. Subsequently, non-hybridized fragments were removed by several washing steps. Details on array data normalization and processing can be found in the Supplemental Experimental Procedures. Illumina methylation data comparing different thymic T cell subsets were downloaded from the NCBI GEO: GSE55111.

### ms-qPCR

The DNA sample (0.5 μg of 5 ALK+ frozen tumors, 6 CD3^+^ T cells, 23 ALK+ paraffin-isolated tumors, 3 ALK− paraffin-isolated tumors, ALK+ Karpas-299 cells, M.*Sss*I-treated control DNA [Zymo Research] and unmethylated control DNA [Zymo Research]) were subjected to bisulfite conversion with the EZ DNA Methylation Kit according to the manufacturer’s protocol. Methylation-specific primers were designed with MethPrimer (http://www.urogene.org/methprimer). Primers are listed in the Supplemental Experimental Procedures. ms-qPCRs were performed according to the MethyLight protocol (Campan et al., 2009) but using SYBR green instead of TaqMan probes for quantification. PMR (percentage of methylated reference) values were calculated according to the following formula: 100 × [(*Gene-X* mean value)_sample_ / (*Alu* mean value)_sample_]/[(*Gene-X* mean value)_M.SssI_ / (*Alu* mean value)_M.SssI_].

### Gene Expression Analysis

Gene expression data from ALCL tumors and normal T cells were obtained from GEO: GSE14879.

### ChIP and qPCR

ChIP was performed as described (Gal-Yam et al., 2008). Approximately 100 μg of chromatin isolated from 10^7^ Karpas-299 or SU-DHL-1 cells were incubated with 50 μL of Dynabeads proteinG (Life Technologies) for 1 hr at 4°C and then with 10 μg of anti-JUNB (Santa Cruz, sc-73X) or 1 μg of anti-histone H3 (Abcam, ab1791), anti-H3K4me3 (Abcam, ab8580), anti-H3K9me3 (Abcam, ab8898), anti-H3K27me3 (Abcam, ab6002), or control immunoglobulin G (IgG) (Santa Cruz, sc-2027) overnight. Bound chromatin was washed, eluted, and de-cross-linked, and DNA was isolated by phenol-chloroform extraction after protein digestion. Primer sequences are listed in the Supplemental Experimental Procedures.

### Knockdown of JunB by shRNA

Knockdown experiments were performed using lentivirus-mediated transduction of Karpas-299 with shRNA vectors as detailed in the Supplemental Experimental Procedures.

## Supplementary Material

Supplemental Information includes Supplemental Experimental Procedures, eight figures, and four tables and can be found with this article online at http://dx.doi.org/10.1016/j.celrep.2016.09.018.

Supplemental Information

## Highlights

- ALK+ and ALK− ALCL show highly similar genome-wide DNA methylation
- ALCL tumor cells closely resemble undifferentiated thymic progenitor cells
- Differentially methylated genes are implicated in major T cell functions
- Oncogenic transcription factors produce epigenetic footprints

## References

[R1] Akimzhanov A, Krenacs L, Schlegel T, Klein-Hessling S, Bagdi E, Stelkovics E, Kondo E, Chuvpilo S, Wilke P, Avots A (2008). Epigenetic changes and suppression of the nuclear factor of activated T cell 1 (NFATC1) promoter in human lymphomas with defects in immunoreceptor signaling. Am J Pathol.

[R2] Al-Hashmi I, Decoteau J, Gruss HJ, Zielenska M, Thorner P, Poon A, Reis M, Freedman M, Lorenzana A (2001). Establishment of a cytokine-producing anaplastic large-cell lymphoma cell line containing the t(2;5) translocation: potential role of cytokines in clinical manifestations. Leuk Lymphoma.

[R3] Alegre ML, Frauwirth KA, Thompson CB (2001). T-cell regulation by CD28 and CTLA-4. Nat Rev Immunol.

[R4] Ambrogio C, Martinengo C, Voena C, Tondat F, Riera L, di Celle PF, Inghirami G, Chiarle R (2009). NPM-ALK oncogenic tyrosine kinase controls T-cell identity by transcriptional regulation and epigenetic silencing in lymphoma cells. Cancer Res.

[R5] Arts RJ, Joosten LA, van der Meer JW, Netea MG (2013). TREM-1: intracellular signaling pathways and interaction with pattern recognition receptors. J Leukoc Biol.

[R6] Bailey TL, Boden M, Buske FA, Frith M, Grant CE, Clementi L, Ren J, Li WW, Noble WS (2009). MEME SUITE: tools for motif discovery and searching. Nucleic Acids Res.

[R7] Benharroch D, Meguerian-Bedoyan Z, Lamant L, Amin C, Brugières L, Terrier-Lacombe MJ, Haralambieva E, Pulford K, Pileri S, Morris SW (1998). ALK-positive lymphoma: a single disease with a broad spectrum of morphology. Blood.

[R8] Berman BP, Weisenberger DJ, Aman JF, Hinoue T, Ramjan Z, Liu Y, Noushmehr H, Lange CP, van Dijk CM, Tollenaar RA (2011). Regions of focal DNA hypermethylation and long-range hypomethylation in colorectal cancer coincide with nuclear lamina-associated domains. Nat Genet.

[R9] Bernstein BE, Birney E, Dunham I, Green ED, Gunter C, Snyder M, ENCODE Project Consortium (2012). An integrated encyclopedia of DNA elements in the human genome. Nature.

[R10] Bischof D, Pulford K, Mason DY, Morris SW (1997). Role of the nucleophosmin (NPM) portion of the non-Hodgkin’s lymphoma-associated NPM-anaplastic lymphoma kinase fusion protein in oncogenesis. Mol Cell Biol.

[R11] Boi M, Zucca E, Inghirami G, Bertoni F (2015). Advances in understanding the pathogenesis of systemic anaplastic large cell lymphomas. Br J Haematol.

[R12] Bouchon A, Dietrich J, Colonna M (2000). Cutting edge: inflammatory responses can be triggered by TREM-1, a novel receptor expressed on neutrophils and monocytes. J Immunol.

[R13] Campan M, Weisenberger DJ, Trinh B, Laird PW (2009). MethyLight. Methods Mol Biol.

[R14] Chiarle R, Gong JZ, Guasparri I, Pesci A, Cai J, Liu J, Simmons WJ, Dhall G, Howes J, Piva R, Inghirami G (2003). NPM-ALK transgenic mice spontaneously develop T-cell lymphomas and plasma cell tumors. Blood.

[R15] Crescenzo R, Abate F, Lasorsa E, Tabbo’ F, Gaudiano M, Chiesa N, Di Giacomo F, Spaccarotella E, Barbarossa L, Ercole E, European T-Cell Lymphoma Study Group, T-Cell Project: Prospective Collection of Data in Patients with Peripheral T-Cell Lymphoma and the AIRC 5xMille Consortium “Genetics-Driven Targeted Management of Lymphoid Malignancies” (2015). Convergent mutations and kinase fusions lead to oncogenic STAT3 activation in anaplastic large cell lymphoma. Cancer Cell.

[R16] Eckerle S, Brune V, Döring C, Tiacci E, Bohle V, Sundström C, Kodet R, Paulli M, Falini B, Klapper W (2009). Gene expression profiling of isolated tumour cells from anaplastic large cell lymphomas: insights into its cellular origin, pathogenesis and relation to Hodgkin lymphoma. Leukemia.

[R17] Egger G, Liang G, Aparicio A, Jones PA (2004). Epigenetics in human disease and prospects for epigenetic therapy. Nature.

[R18] ENCODE Project Consortium (2012). An integrated encyclopedia of DNA elements in the human genome. Nature.

[R19] Fernandez AF, Assenov Y, Martin-Subero JI, Balint B, Siebert R, Taniguchi H, Yamamoto H, Hidalgo M, Tan AC, Galm O (2012). A DNA methylation fingerprint of 1628 human samples. Genome Res.

[R20] Ford JW, McVicar DW (2009). TREM and TREM-like receptors in inflammation and disease. Curr Opin Immunol.

[R21] Fujimoto J, Shiota M, Iwahara T, Seki N, Satoh H, Mori S, Yamamoto T (1996). Characterization of the transforming activity of p80, a hyperphosphorylated protein in a Ki-1 lymphoma cell line with chromosomal translocation t(2;5). Proc Natl Acad Sci USA.

[R22] Gal-Yam EN, Egger G, Iniguez L, Holster H, Einarsson S, Zhang X, Lin JC, Liang G, Jones PA, Tanay A (2008). Frequent switching of Polycomb repressive marks and DNA hypermethylation in the PC3 prostate cancer cell line. Proc Natl Acad Sci USA.

[R23] Greer JP, Kinney MC, Collins RD, Salhany KE, Wolff SN, Hainsworth JD, Flexner JM, Stein RS (1991). Clinical features of 31 patients with Ki-1 anaplastic large-cell lymphoma. J Clin Oncol.

[R24] Halachev K, Bast H, Albrecht F, Lengauer T, Bock C (2012). EpiExplorer: live exploration and global analysis of large epigenomic datasets. Genome Biol.

[R25] Huang DW, Sherman BT, Lempicki RA (2009a). Bioinformatics enrichment tools: paths toward the comprehensive functional analysis of large gene lists. Nucleic Acids Res.

[R26] Huang DW, Sherman BT, Lempicki RA (2009b). Systematic and integrative analysis of large gene lists using DAVID bioinformatics resources. Nat Protoc.

[R27] Iqbal J, Weisenburger DD, Greiner TC, Vose JM, McKeithan T, Kucuk C, Geng H, Deffenbacher K, Smith L, Dybkaer K, International Peripheral T-Cell Lymphoma Project (2010). Molecular signatures to improve diagnosis in peripheral T-cell lymphoma and prognostication in angioimmunoblastic T-cell lymphoma. Blood.

[R28] Ji H, Ehrlich LI, Seita J, Murakami P, Doi A, Lindau P, Lee H, Aryee MJ, Irizarry RA, Kim K (2010). Comprehensive methylome map of lineage commitment from haematopoietic progenitors. Nature.

[R29] Jones PA, Wolkowicz MJ, Rideout WM, Gonzales FA, Marziasz CM, Coetzee GA, Tapscott SJ (1990). De novo methylation of the MyoD1 CpG island during the establishment of immortal cell lines. Proc Natl Acad Sci USA.

[R30] Kollmann K, Heller G, Ott RG, Scheicher R, Zebedin-Brandl E, Schneckenleithner C, Simma O, Warsch W, Eckelhart E, Hoelbl A (2011). c-JUN promotes BCR-ABL-induced lymphoid leukemia by inhibiting methylation of the 5′ region of Cdk6. Blood.

[R31] Laimer D, Dolznig H, Kollmann K, Vesely PW, Schlederer M, Merkel O, Schiefer AI, Hassler MR, Heider S, Amenitsch L (2012). PDGFR blockade is a rational and effective therapy for NPM-ALK-driven lymphomas. Nat Med.

[R32] Lamant L, de Reyniès A, Duplantier MM, Rickman DS, Sabourdy F, Giuriato S, Brugières L, Gaulard P, Espinos E, Delsol G (2007). Gene-expression profiling of systemic anaplastic large-cell lymphoma reveals differences based on ALK status and two distinct morphologic ALK+ subtypes. Blood.

[R33] Laurent C, Lopez C, Desjobert C, Berrebi A, Damm-Welk C, Delsol G, Brousset P, Lamant L (2012). Circulating t(2;5)-positive cells can be detected in cord blood of healthy newborns. Leukemia.

[R34] Lehnertz B, Ueda Y, Derijck AA, Braunschweig U, Perez-Burgos L, Kubicek S, Chen T, Li E, Jenuwein T, Peters AH (2003). Suv39h-mediated histone H3 lysine 9 methylation directs DNA methylation to major satellite repeats at pericentric heterochromatin. Curr Biol.

[R35] Leventaki V, Drakos E, Medeiros LJ, Lim MS, Elenitoba-Johnson KS, Claret FX, Rassidakis GZ (2007). NPM-ALK oncogenic kinase promotes cell-cycle progression through activation of JNK/cJun signaling in anaplastic large-cell lymphoma. Blood.

[R36] Li P, Burke S, Wang J, Chen X, Ortiz M, Lee SC, Lu D, Campos L, Goulding D, Ng BL (2010). Reprogramming of T cells to natural killer-like cells upon Bcl11b deletion. Science.

[R37] Machanick P, Bailey TL (2011). MEME-ChIP: motif analysis of large DNA datasets. Bioinformatics.

[R38] Malcolm TI, Villarese P, Fairbairn CJ, Lamant L, Trinquand A, Hook CE, Burke GA, Brugières L, Hughes K, Payet D (2016). Anaplastic large cell lymphoma arises in thymocytes and requires transient TCR expression for thymic egress. Nat Commun.

[R39] Martin-Subero JI, Ammerpohl O, Bibikova M, Wickham-Garcia E, Agirre X, Alvarez S, Brüggemann M, Bug S, Calasanz MJ, Deckert M (2009). A comprehensive microarray-based DNA methylation study of 367 hematological neoplasms. PLoS ONE.

[R40] Mathas S, Hinz M, Anagnostopoulos I, Krappmann D, Lietz A, Jundt F, Bommert K, Mechta-Grigoriou F, Stein H, Dörken B, Scheidereit C (2002). Aberrantly expressed c-Jun and JunB are a hallmark of Hodgkin lymphoma cells, stimulate proliferation and synergize with NF-kappa B. EMBO J.

[R41] Morris SW, Kirstein MN, Valentine MB, Dittmer KG, Shapiro DN, Saltman DL, Look AT (1994). Fusion of a kinase gene, ALK, to a nucleolar protein gene, NPM, in non-Hodgkin’s lymphoma. Science.

[R42] Moti N, Malcolm T, Hamoudi R, Mian S, Garland G, Hook CE, Burke GA, Wasik MA, Merkel O, Kenner L (2015). Anaplastic large cell lymphoma-propagating cells are detectable by side population analysis and possess an expression profile reflective of a primitive origin. Oncogene.

[R43] Nagasawa T, Zhang Q, Raghunath PN, Wong HY, El-Salem M, Szallasi A, Marzec M, Gimotty P, Rook AH, Vonderheid EC (2006). Multigene epigenetic silencing of tumor suppressor genes in T-cell lymphoma cells; delayed expression of the p16 protein upon reversal of the silencing. Leuk Res.

[R44] Ohm JE, McGarvey KM, Yu X, Cheng L, Schuebel KE, Cope L, Mohammad HP, Chen W, Daniel VC, Yu W (2007). A stem cell-like chromatin pattern may predispose tumor suppressor genes to DNA hypermethylation and heritable silencing. Nat Genet.

[R45] Okamura RM, Sigvardsson M, Galceran J, Verbeek S, Clevers H, Grosschedl R (1998). Redundant regulation of T cell differentiation and TCRalpha gene expression by the transcription factors LEF-1 and TCF-1. Immunity.

[R46] Piazza R, Magistroni V, Mogavero A, Andreoni F, Ambrogio C, Chiarle R, Mologni L, Bachmann PS, Lock RB, Collini P (2013). Epigenetic silencing of the proapoptotic gene BIM in anaplastic large cell lymphoma through an MeCP2/SIN3a deacetylating complex. Neoplasia.

[R47] Piva R, Pellegrino E, Mattioli M, Agnelli L, Lombardi L, Boccalatte F, Costa G, Ruggeri BA, Cheng M, Chiarle R (2006). Functional validation of the anaplastic lymphoma kinase signature identifies CEBPB and BCL2A1 as critical target genes. J Clin Invest.

[R48] Popnikolov NK, Payne DA, Hudnall SD, Hawkins HK, Kumar M, Norris BA, Elghetany MT (2000). CD13-positive anaplastic large cell lymphoma of T-cell origin—a diagnostic and histogenetic problem. Arch Pathol Lab Med.

[R49] Rassidakis GZ, Feretzaki M, Atwell C, Grammatikakis I, Lin Q, Lai R, Claret FX, Medeiros LJ, Amin HM (2005). Inhibition of Akt increases p27Kip1 levels and induces cell cycle arrest in anaplastic large cell lymphoma. Blood.

[R50] Rodriguez RM, Suarez-Alvarez B, Mosén-Ansorena D, García-Peydró M, Fuentes P, García-León MJ, Gonzalez-Lahera A, Macias-Camara N, Toribio ML, Aransay AM, Lopez-Larrea C (2015). Regulation of the transcriptional program by DNA methylation during human αβ T-cell development. Nucleic Acids Res.

[R51] Scarfò I, Pellegrino E, Mereu E, Kwee I, Agnelli L, Bergaggio E, Garaffo G, Vitale N, Caputo M, Machiorlatti R, European T-Cell Lymphoma Study Group (2016). Identification of a new subclass of ALK-negative ALCL expressing aberrant levels of ERBB4 transcripts. Blood.

[R52] Schlesinger Y, Straussman R, Keshet I, Farkash S, Hecht M, Zimmerman J, Eden E, Yakhini Z, Ben-Shushan E, Reubinoff BE (2007). Polycomb-mediated methylation on Lys27 of histone H3 pre-marks genes for de novo methylation in cancer. Nat Genet.

[R53] Shen H, Laird PW (2013). Interplay between the cancer genome and epigenome. Cell.

[R54] Staber PB, Vesely P, Haq N, Ott RG, Funato K, Bambach I, Fuchs C, Schauer S, Linkesch W, Hrzenjak A (2007). The oncoprotein NPM-ALK of anaplastic large-cell lymphoma induces JUNB transcription via ERK1/2 and JunB translation via mTOR signaling. Blood.

[R55] Stein H, Mason DY, Gerdes J, O’Connor N, Wainscoat J, Pallesen G, Gatter K, Falini B, Delsol G, Lemke H (1985). The expression of the Hodgkin’s disease associated antigen Ki-1 in reactive and neoplastic lymphoid tissue: evidence that Reed-Sternberg cells and histiocytic malignancies are derived from activated lymphoid cells. Blood.

[R56] Swerdlow SH, Campo E, Harris NL, Jaffe ES, Pileri SA, Stein H, Thiele J, Vardiman JW (2008). WHO Classification of Tumours of Haematopoietic and Lymphoid Tissues.

[R57] Tachibana M, Matsumura Y, Fukuda M, Kimura H, Shinkai Y (2008). G9a/GLP complexes independently mediate H3K9 and DNA methylation to silence transcription. EMBO J.

[R58] Teschendorff AE, Menon U, Gentry-Maharaj A, Ramus SJ, Weisenberger DJ, Shen H, Campan M, Noushmehr H, Bell CG, Maxwell AP (2010). Age-dependent DNA methylation of genes that are suppressed in stem cells is a hallmark of cancer. Genome Res.

[R59] Tivol EA, Borriello F, Schweitzer AN, Lynch WP, Bluestone JA, Sharpe AH (1995). Loss of CTLA-4 leads to massive lymphoproliferation and fatal multiorgan tissue destruction, revealing a critical negative regulatory role of CTLA-4. Immunity.

[R60] Turner SD, Tooze R, Maclennan K, Alexander DR (2003). Vav-promoter regulated oncogenic fusion protein NPM-ALK in transgenic mice causes B-cell lymphomas with hyperactive Jun kinase. Oncogene.

[R61] Turner SD, Yeung D, Hadfield K, Cook SJ, Alexander DR (2007). The NPM-ALK tyrosine kinase mimics TCR signalling pathways, inducing NFAT and AP-1 by RAS-dependent mechanisms. Cell Signal.

[R62] Viré E, Brenner C, Deplus R, Blanchon L, Fraga M, Didelot C, Morey L, Van Eynde A, Bernard D, Vanderwinden JM (2006). The Polycomb group protein EZH2 directly controls DNA methylation. Nature.

[R63] Waterhouse P, Penninger JM, Timms E, Wakeham A, Shahinian A, Lee KP, Thompson CB, Griesser H, Mak TW (1995). Lymphoproliferative disorders with early lethality in mice deficient in Ctla-4. Science.

[R64] Widschwendter M, Fiegl H, Egle D, Mueller-Holzner E, Spizzo G, Marth C, Weisenberger DJ, Campan M, Young J, Jacobs I, Laird PW (2007). Epigenetic stem cell signature in cancer. Nat Genet.

[R65] Yui MA, Rothenberg EV (2014). Developmental gene networks: a triathlon on the course to T cell identity. Nat Rev Immunol.

[R66] Zamo A, Chiarle R, Piva R, Howes J, Fan Y, Chilosi M, Levy DE, Inghirami G (2002). Anaplastic lymphoma kinase (ALK) activates Stat3 and protects hematopoietic cells from cell death. Oncogene.

[R67] Zhang Q, Wang HY, Marzec M, Raghunath PN, Nagasawa T, Wasik MA (2005). STAT3- and DNA methyltransferase 1-mediated epigenetic silencing of SHP-1 tyrosine phosphatase tumor suppressor gene in malignant T lymphocytes. Proc Natl Acad Sci USA.

[R68] Zhang Q, Wang HY, Liu X, Wasik MA (2007). STAT5A is epigenetically silenced by the tyrosine kinase NPM1-ALK and acts as a tumor suppressor by reciprocally inhibiting NPM1-ALK expression. Nat Med.

[R69] Zhang Q, Wang HY, Bhutani G, Liu X, Paessler M, Tobias JW, Baldwin D, Swaminathan K, Milone MC, Wasik MA (2009). Lack of TNFalpha expression protects anaplastic lymphoma kinase-positive T-cell lymphoma (ALK+ TCL) cells from apoptosis. Proc Natl Acad Sci USA.

[R70] Zhang Q, Wang HY, Liu X, Bhutani G, Kantekure K, Wasik M (2011). IL-2R common gamma-chain is epigenetically silenced by nucleophosphin-anaplastic lymphoma kinase (NPM-ALK) and acts as a tumor suppressor by targeting NPM-ALK. Proc Natl Acad Sci USA.

