# Supplementary material for: Insights into the Pathogenesis of Anaplastic Large-Cell Lymphoma through Genome-wide DNA Methylation Profiling
Source: Cell Rep. Author manuscript; Available in PMC 2018 Jul 30. (PMC6066089; doi:10.1016/j.celrep.2016.09.018)
Supplement: Supplemental Information [file NIHMS70902-supplement-Supplemental_Information.pdf]

Cell Reports, Volume *17*

Supplemental Information

Insights into the Pathogenesis of Anaplastic Large-Cell Lymphoma through Genome-wide DNA Methylation Profiling

Melanie R. Hassler, Walter Pulverer, Ranjani Lakshminarasimhan, Elisa Redl, Julia Hacker, Gavin D. Garland, Olaf Merkel, Ana-Iris Schiefer, Ingrid Simonitsch-Klupp, Lukas Kenner, Daniel J. Weisenberger, Andreas Weinhaeusel, Suzanne D. Turner, and Gerda Egger

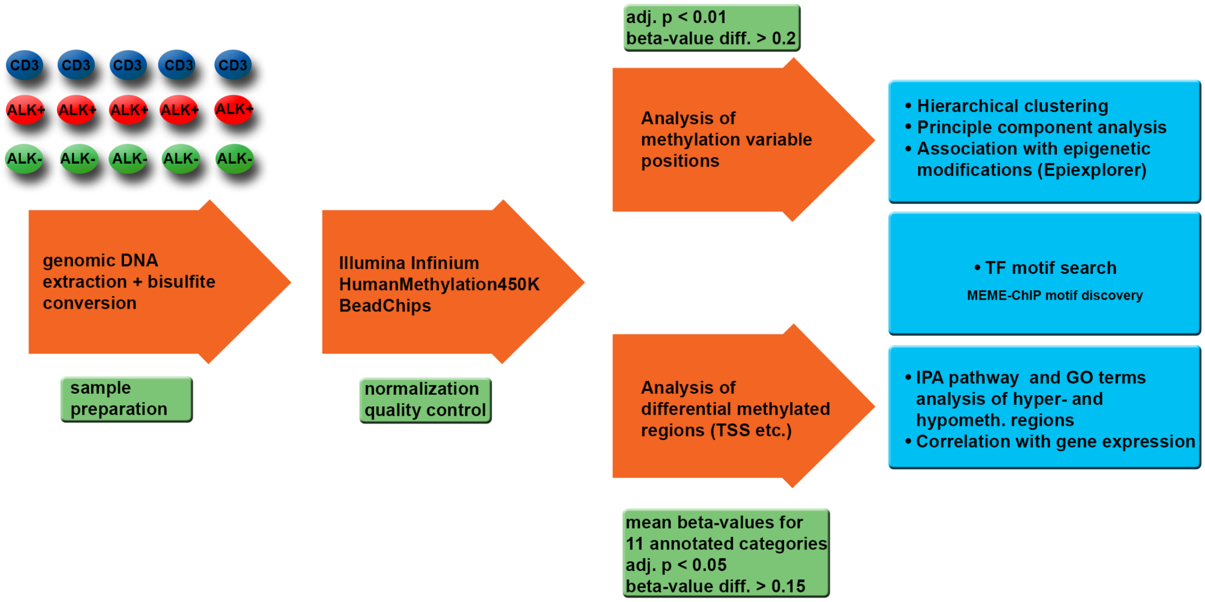

Figure S1. Flow chart of ALK+ and ALK- ALCL methylation analyses, related to Figure 1. Diagram illustrating the workflow from sample preparation, processing and bioinformatic analysis.

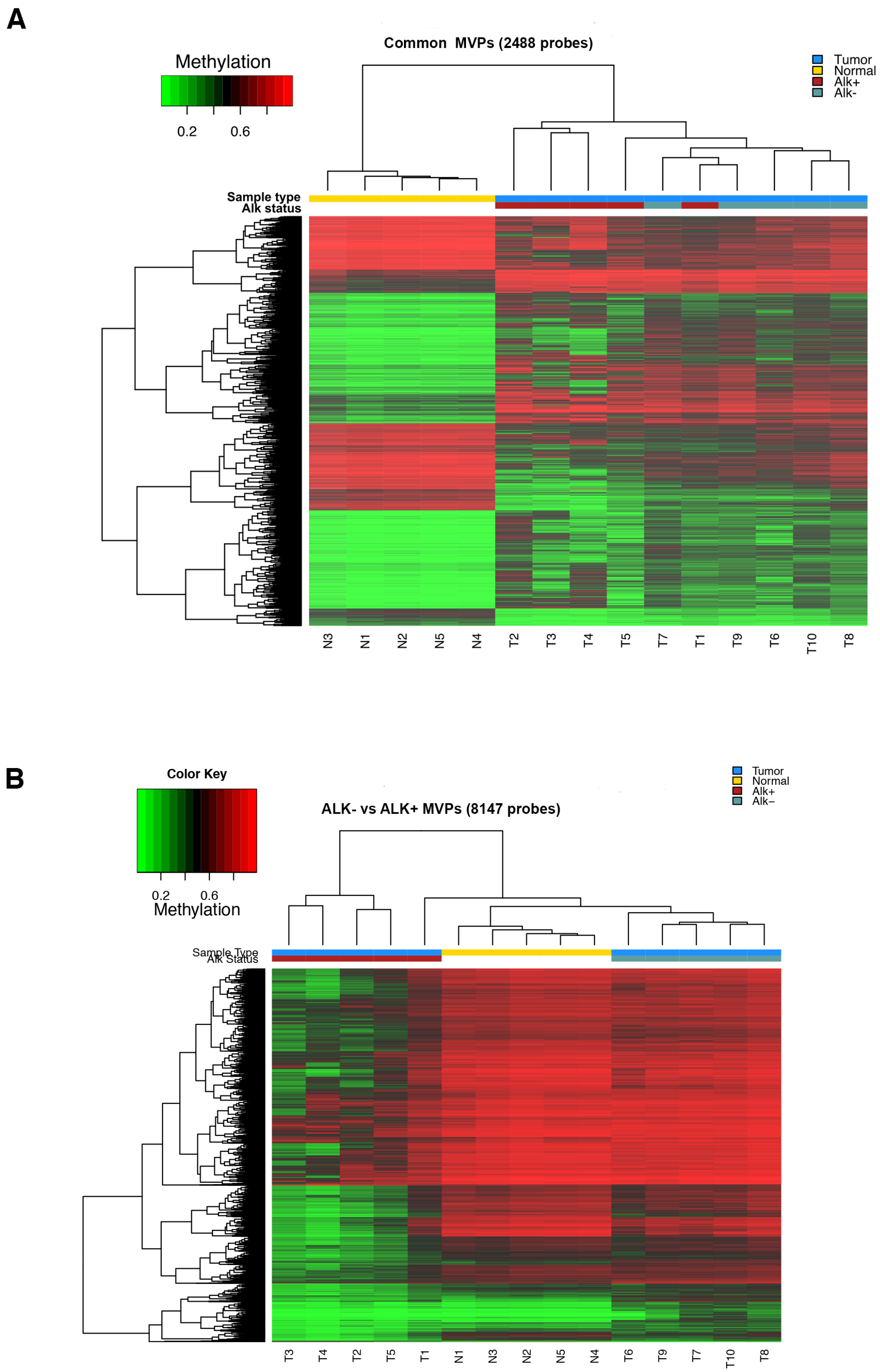

Figure S2. Hierarchical clustering of ALK+ and ALK- ALCL compared to normal, related to Figure 1. (**A**) Hierarchical clustering of common MVPs in ALK+ and ALK- ALCL compared to normal $CD3^+$ cells shows separation into tumor versus normal samples. (**B**) Clustering based on the 8147 differentially methylated probes between ALK- and ALK+ locates ALK- closer to $CD3^+$ T cells.

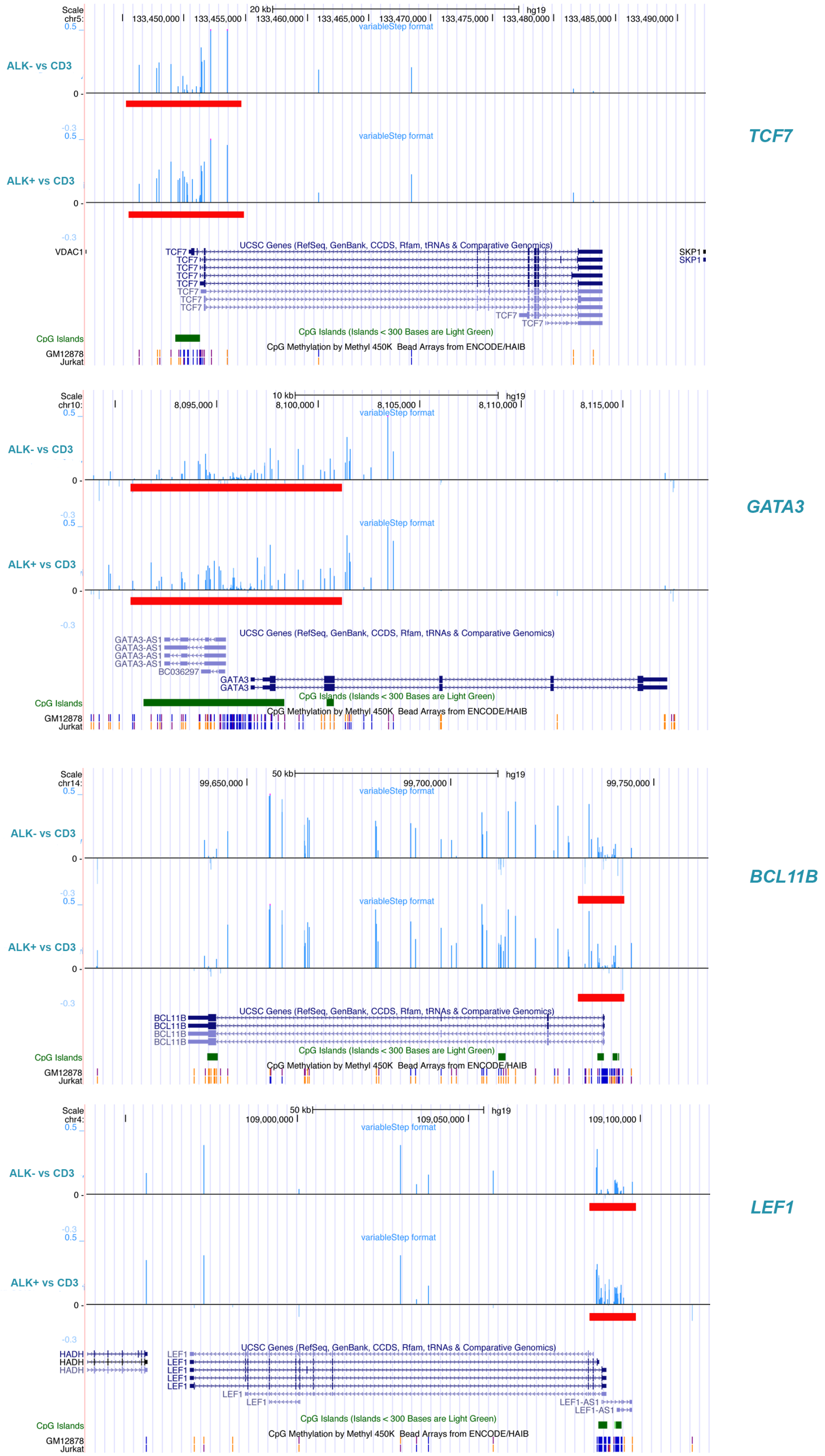

Figure S3. Hypermethylation of T cell specific transcription factors, related to Figure 3. UCSC genome browser tracks showing hypermethylation of indicated transcription factors (blue tracks on top indicate differential methylation (ß value difference) between ALK+ or ALK- ALCL tumors and $CD3^{+}$ T cells; red bars, promoter regions of individual genes; green bars, CpG islands; dark blue, UCSC genes).

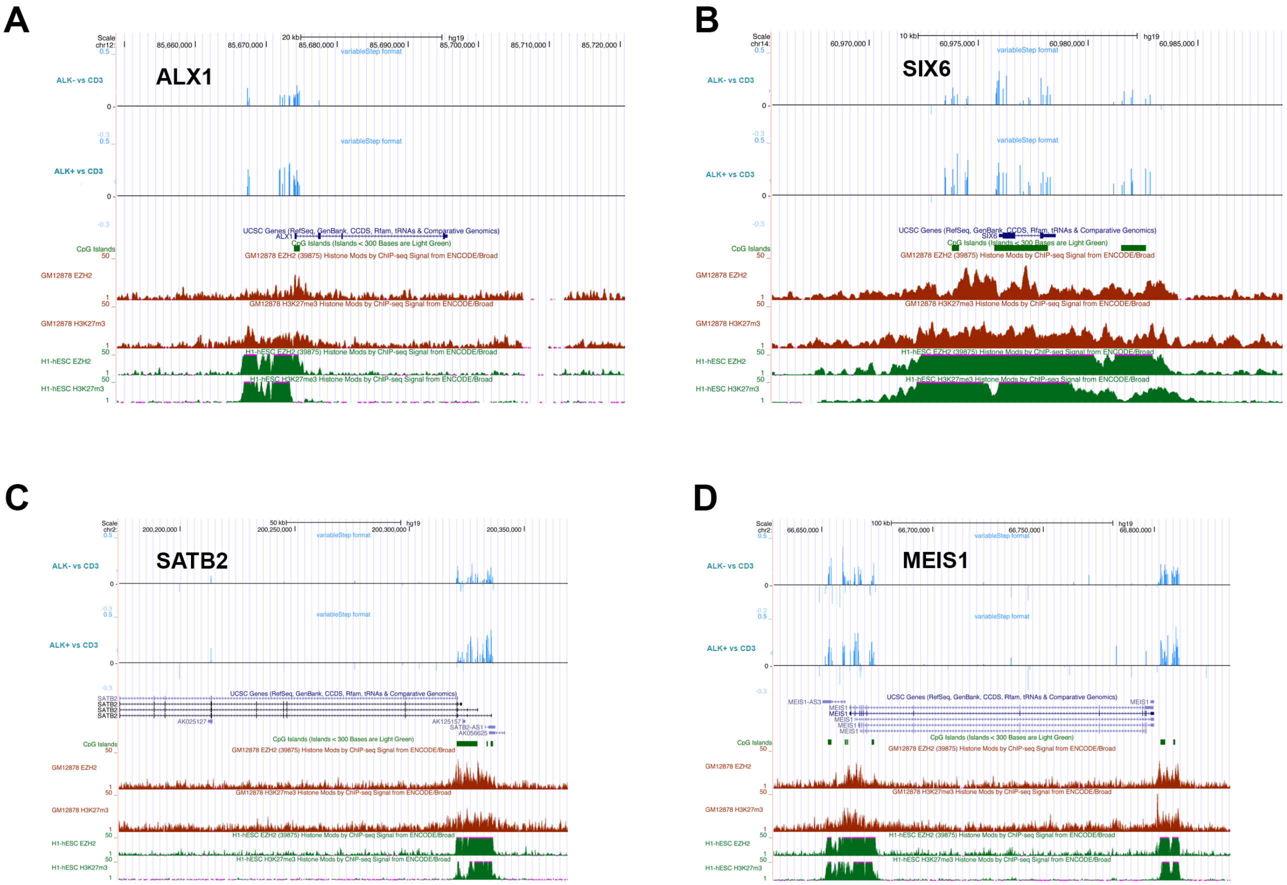

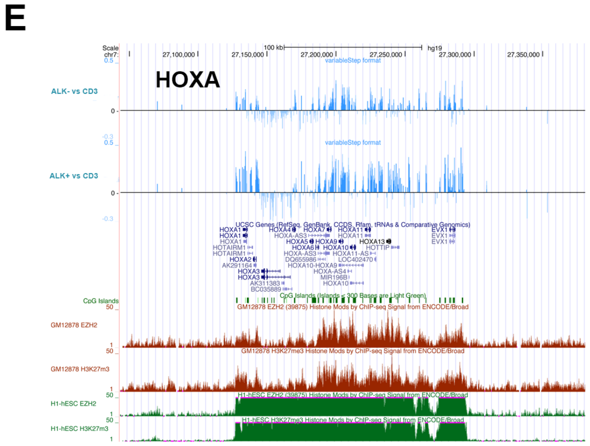

F

Gene Symbol	Name
ALX1	ALX homeobox 1
BARHL2	BarH-like homeobox 2
NKX6-2	NK6 homeobox 2
POU3F3	POU class 3 homeobox 3
SATB2	SATB homeobox 2
SIX6	SIX homeobox 6
TLX3	T-cell leukemia homeobox 3
DLX6	distal-less homeobox 6
EN1	engrailed homeobox 1
GBX2	gastrulation brain homeobox 2
GSC	goosecoid homeobox
HOXA7	homeobox A7
HOXD4	homeobox D4
IRX1	iroquois homeobox 1
IRX2	iroquois homeobox 2
MEOX2	mesenchyme homeobox 2
MSX1	msh homeobox 1
PHOX2A	paired-like homeobox 2a
PROX1	prospero homeobox 1

Figure S4. Epigenetic switching of homeobox genes, related to Figure 4.
(A-E) UCSC browser graphics showing ALK+ and ALK- ALCL hypermethylation as compared to $CD3^+$ T cells (blue track, top), UCSC genes (blue), CpG island location (green bar) and ChIP Seq data on EZH2 and H3K27me3 occupancy in lymphoblastoid (GM12878, red) and ESC (green) as visualized from ENCODE data. (F) Table listing homeobox genes that show epigenetic switching.

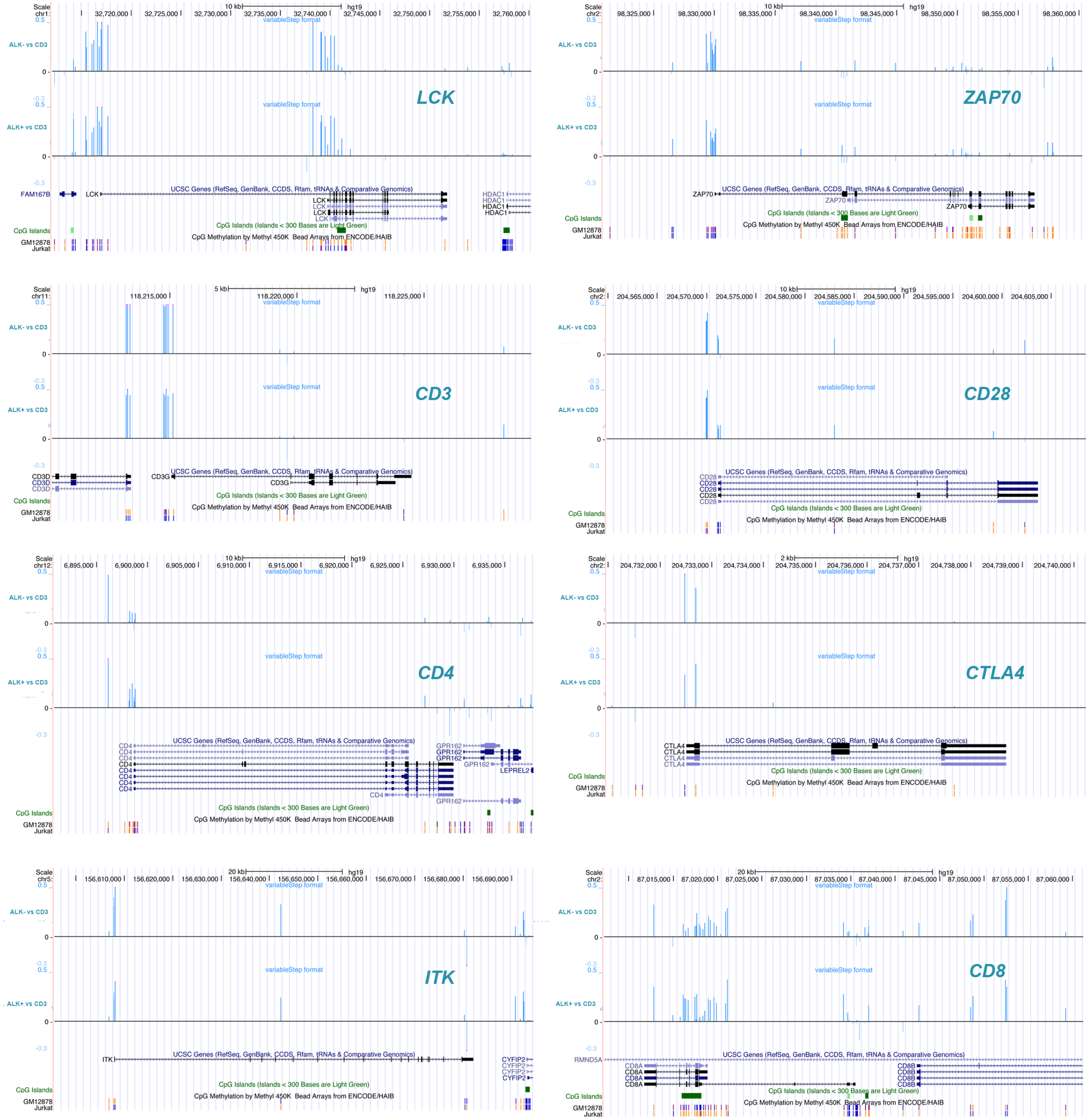

Figure S5. Methylation of TCR pathway genes, related to Figure 6. UCSC browser graphic illustrating DNA methylation differences in ALK+ and ALK- ALCL versus CD3^{+} T cells. Blue track indicates differential DNA methylation of indicated genes. Green boxes indicate CpG islands. Lowest track shows ENCODE methylation data of lymphoblastoid (GM12878) and Jurkat T cell leukemia cell lines (orange indicates high methylation, purple medium and blue low methylation of respective CpG sites).

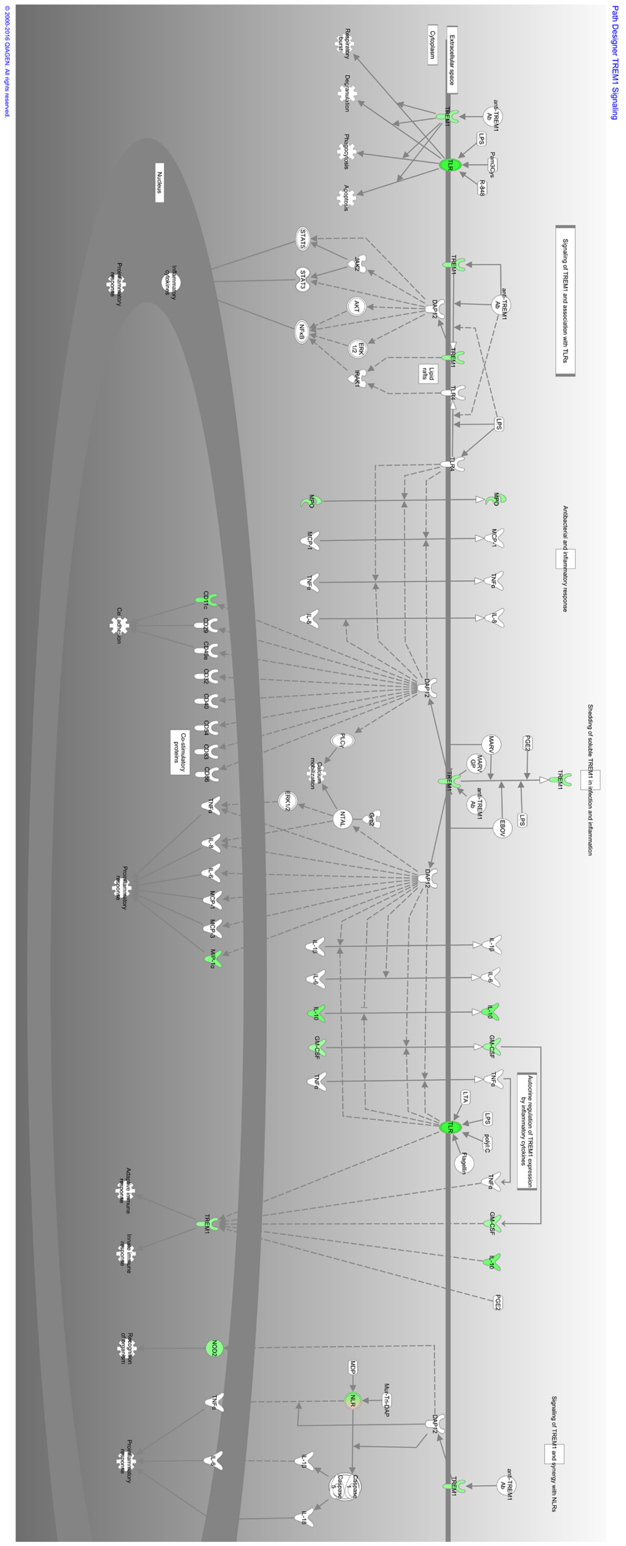

Figure S6. TREM1 signaling as top pathway associated with hypomethylated promoters, related to Figure 6. TREM1 signaling pathways and hypomethylated promoters (green, genes affected by DNA hypomethylation in ALCL tumors). The pathways were generated through the use of QIAGEN's Ingenuity Pathway Analysis (IPA®,QIAGEN Redwood City, www.qiagen.com/ingenuity).

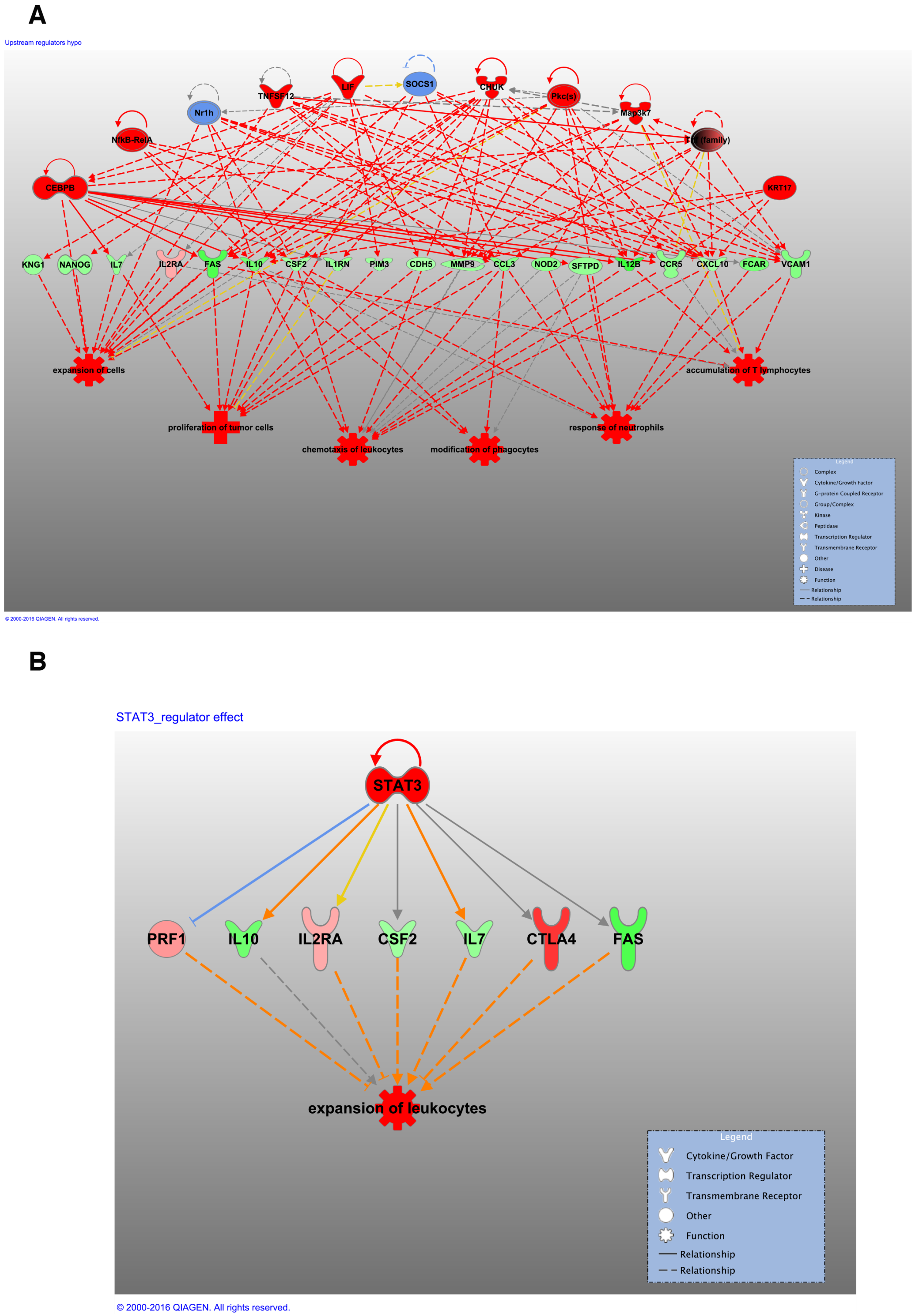

Figure S7. Networks and network regulators related to top hyper- or hypomethylated genes contain cytokines and genes involved in inflammatory responses, related to Figure 6. (A) Upstream regulators and downstream functional connections of hypomethylated genes including cytokines and receptors relevant in ALCL. **(B)** STAT3 regulator effect on cytokines and receptors involved in leukocyte regulation. The networks were generated through the use of QIAGEN's Ingenuity Pathway Analysis (IPA®,QIAGEN Redwood City, www.qiagen.com/ingenuity).

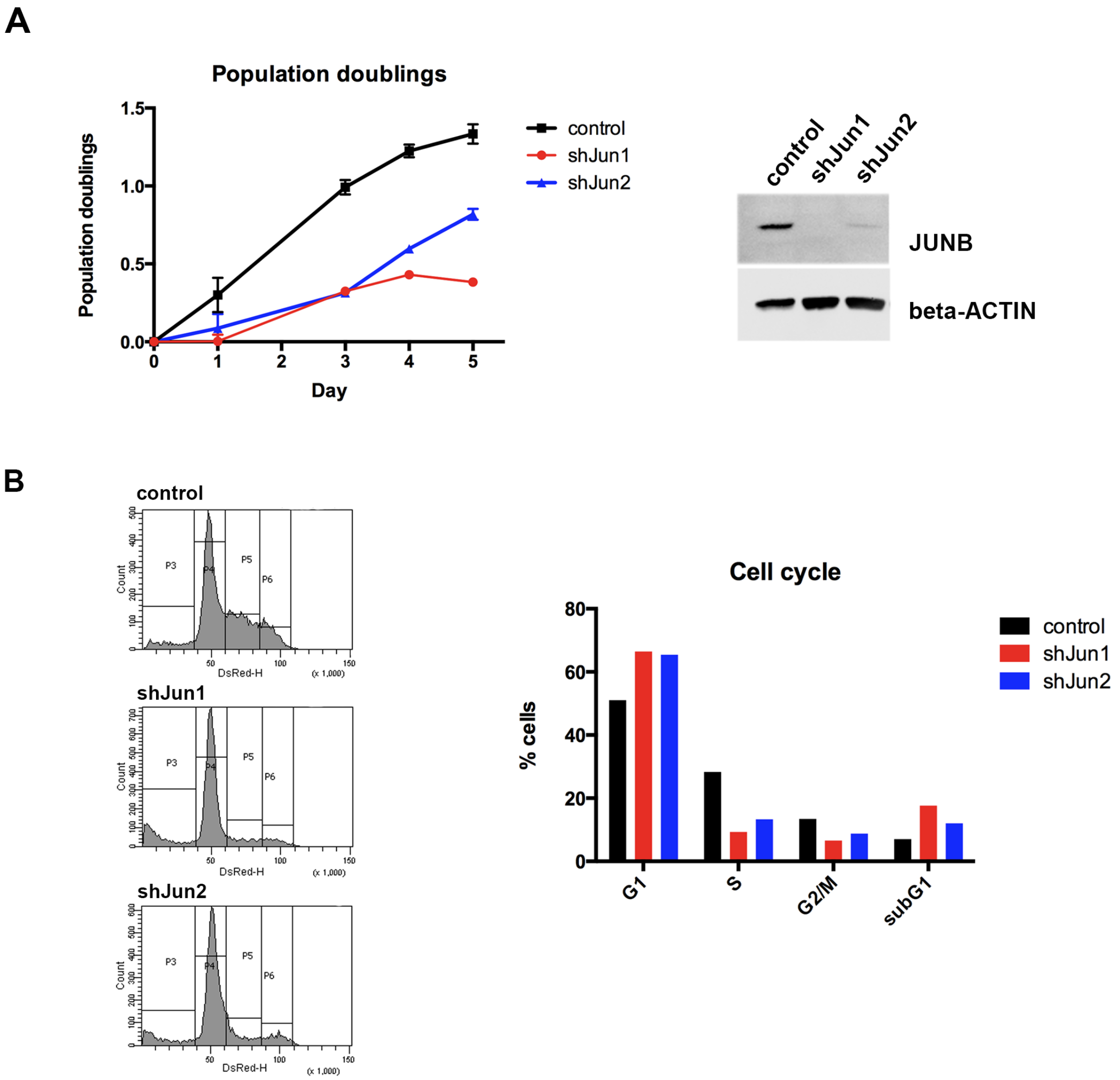

Figure S8. Knock-down of AP1 transcription factor JUNB in ALK+ ALCL cells, related to Figure 6. (**A**) (left) Cell population doublings of Karpas-299 cells after sh-mediated knockdown of JUNB. Two independent sh-RNAs were used for the knockdown (shJun1 and shJun2, specific oligo targeting JUNB; control, scrambled oligo). Values are means ± SD. Each value is the mean of two replicates. (right) Western blot showing JUNB levels in Karpas-299 cells after JUNB knockdown. Beta-ACTIN was used as a loading control. (**B**) (left) Representative cell cycle analyses of PI stained Karpas-299 cells after JUNB knockdown or control cells by FACS (sh control, Karpas-299 cells infected with control oligo vector; shJun1/shJun2, Karpas-299 cells infected with JUNB targeting oligo). (right) Representative cell cycle distribution of Karpas-299 cells after JUNB knockdown or control cells divided into percentage of cells in different cell cycle phases. Sub G1 represents apoptotic cells.

Table S1. Significant GO Terms associated with hypo- and hypermethylated promoters in ALK+ and ALK- ALCL tumor samples. Related to Figure 5. For Table see separate file.

up-regulated (>1.5 fold)		
**Hypomethylated DMRs ALK+ (674)**	93	ANKRD22,ACPP,MARCO,CSTA,TK2,HRH1,AIM2,S100A2,MS4A6A,C12orf10,SERPINA1,RTN3,OLFML3,MPEG1,SRPX2,RCAN2,LGALS1,A2M,S100A8,CCL18,S100A10,IL1RN,GAPDH,MGP,PMAIP1,SIGLEC10,ITGAX,PRKCDBP,FAM50B,KSR1,SNAPC5,ARSB,DCPS,CXCL10,SLC35E4,CLU,ARNTL2,CFD,CILP,ITPRIPL2,NCF4,CCDC42B,SFN,HEATR2,TUBB,GYG1,CLCC1,AGPAT2,MCAM,MS4A7,RAMP1,UPP1,COL1A1,PHGDH,LYN,LTB4R,SERPINB8,SUOX,MMP9,PKIG,CD5L,PHLPP1,SYNGR1,PIM3,EIF3G,MTX1,FUCA2,NOL3,MYL4,C1R,DCN,C19orf24,ASXL1,FXYD6,SPATS2,SLC15A4,HLA-DPB1,ANLN

down-regulated (<1.5 fold)		
**Hypermethylated DMRs ALK+ (501)**	65	THEMIS,CD3G,CD2,C14orf64,PPP6C,KCNJ2,CD52,CD7,ABCB4,MGAT4A,LCK,ZNF665,CXCR6,GIMAP1,UBASH3A,CHI3L2,RHOH,CD6,GIMAP5,LMBRD1,PPP1R16B,FAIM3,PSD4,DRD5,TBC1D10C,NMT2,THRB,LPIN2,SEMA4D,LAG3,LRMP,RUNDC3B,FAM102A,TMIGD2,SLIT2,ASXL3,CXCR5,LY9,AKNA,NDFIP1,SLC40A1,RGS1,ITGAL,CD47,RCSD1,ZNF101,STK38,KLF3,JAZF1,P4HA3,TLR5,CD48

up-regulated (>1.5 fold)		
**Hypomethylated DMRs ALK- (468)**	55	MPEG1,CSTA,GAPDH, HLA-DQA1, SPAG4, IL23R, CLIC2, ADORA3, BLK, LGALS1,TRAPPC3,RTN3,NCEH1,VCAM1,ANK,RD29,TYROBP,PLA2G4C,MGP,PCBD1,ITPRIPL2,CTSZ,ST7,SLC35E4,MMP9,DCPS,TJP2, MGST2, RALGPS1, BCAT1, ABI3BP, ROBO4,MS4A6A,CXCL10,LANCL2,CD59,PRKCDBP, TMEM119,A2M,CD86,CD58,HLA-DQB2,TUBB, CTSG, ANKRD22, CCRN4L, C1orf54, IFI30, LMO4, S100A10, APOBEC3B,PHGDH,ADAMDEC1,CCL8,UQCRFS1,TK1

down-regulated (<1.5 fold)		
**Hypermethylated DMRs ALK- (674)**	84	TXK,CTLA4,CD3G,MGAT4A,CXCR6,IL7R,PPP6C,CD7,LCK,UBASH3A,CD28,RIOK3,DENND2D,TMIGD2,GNG2,ITK,RHOH,EVI2A,EXOC1,SLC22A3,APBA2,14orf64,SATB1,SPOCK2,PIK3CG,CHI3L2,LY9,PTPN22,GPR171,PPP1R16B,LHX2,TC2N,FAM102A,LAG3,HIVEP3,ARHGEF10,ATP10A,RIT1,GBP1,FAIM3,ABCB4,LAX1,NMT2,DRD5,CD6,MYO5C,GIMAP7,UNC5D,CD96,SLFN5,CD52,LAPTM5,TNFSF8,SPN,NLRC3,EVL,TMEM71,LCP2,NDFIP1,CD5,NUP210,ARHGAP8,IL27RA,GIMAP5,SLAMF6,SYTL1,GIMAP1,AUTS2,INPP5D,ARHGAP15,RASEF,TSPAN32,TMC8,PLEKHM3,CARD8,CXCR5,C11orf21,TRAF3IP3,ORAI2,ZAP70,ARHGAP9,LRMP,KIAA0754,THRB

Table S2. Genes with inverse correlation between methylation and expression, related to Figure 5.

Table S3. GO Term analysis of differentially methylated promoters inverse correlated with gene expression, related to Figure 5. For table see separate file.

Number	Age	Sex	Ann-Arbor	Morphology	ALK-Status	Therapy	Follow-Up
T8	55	f	IIA	ALCL, classical	negativ	CHOP	complete remission
T9	48	m	IVB	ALCL, classical	negativ	CHOP	dead of disease
T10	67	f	na	ALCL, classical	negativ	na	na
T7	17	f	na	ALCL, classical	negativ	na	relapse
T6	15	f	II	ALCL, classical	negativ	NHL-BFM	complete remission
T1	11	f	IIE	ALCL, classical	positiv	NHL-BFM	complete remission
T2	14	m	na	ALCL, classical	positiv	na	na
T4	8	f	IIE	ALCL, classical	positiv	NHL-BFM	complete remission
T5	16	f	IVA	ALCL, classical	positiv	NHL-BFM 95	complete remission
T3	21	f	IIA	ALCL, classical	positiv	CHOP	complete remission

Table S4. Demographic data of ALCL patients included in the analyses. CHOP, chemoterapy including Cyclophosphamide, Hydroxydaunomycin (Doxorubicin), Oncovin (Vincristinine), Prednisolone; na, data not available; NHL-BFM, patients were enrolled in studies of the Non-Hodgkin-Lymphoma Berlin-Frankfurt-Münster study group and treated according to protocols (https://www.uni-giessen.de/fbz/fb11/nhl-bfm?set_language=en).

SUPPLEMENTAL EXPERIMENTAL PROCEDURES

Isolation of genomic DNA from lymphocytes, frozen tumors and FFPE tissue

DNA isolation from $CD3^+$ lymphocytes, frozen tumors and FFPE tissues was performed with the QIAamp DNA Blood Mini Kit. $CD3^+$ cells were directly lysed with buffer AL (containing 10 mg/ml RNAse, Roche), incubated with protease K and processed according to protocol. Frozen tumors were pulverized in liquid nitrogen, lysed with buffer AL (containing 10 mg/ml RNAse, Roche), incubated with protease K until completely dissolved and processed according to protocol. FFPE tissue was dissected using microarray punches, deparaffinzed with xylol, washed with EtOH, dried and resuspended in buffer ATL. Then, the suspension was incubated with protease K for 3 days and further processed according to protocol.

Human ALK+ ALCL cell lines

Human ALCL cell lines KARPAS-299 and SUDHL1 were obtained from the DSMZ (https://www.dsmz.de) and grown in RPMI 1640 medium (GIBCO) containing 10% FBS (fetal

bovine serum) and 1% penicillin/streptomycin at 37°C in an atmosphere of 5% CO_2 and 95% room air.

Isolation of genomic DNA from cell lines

Cells were dissolved in genomic DNA isolation buffer (0.4M NaCl, 0.2% SDS, 0.1M Tris pH 8.3, 5 mM EDTA). After RNase A (20 µg/ml, Invitrogen) and Proteinase K (500 µg/ml, Invitrogen) digestion, phenol/chloroform extraction was performed and the DNA was precipitated with isopropanol. The DNA pellet was washed and dissolved in sterile water. DNA concentration was measured on a Nanodrop 2000 (Thermo Scientific).

Illumina Infinium HM450 DNA methylation data production

Genomic DNA is first treated with sodium bisulfite using the Zymo EZ DNA Methylation kit (Zymo Research, Irvine, CA) according to the manufacturer's instructions. We assessed the amount of bisulfite-converted DNA as well as the completeness of bisuflite conversion for each sample using a panel of MethyLight-based real-time PCR quality control assays as described previously (PMID: 18987824). After bisulfite conversion, each sample is whole genome amplified (WGA) and then enzymatically fragmented. Samples are then hybridized overnight to a 12 sample BeadChip, in which the WGA-DNA molecules anneal to locus-specific DNA oligomers linked to individual bead types. The oligomer probe designs follow the Infinium I and II chemistries, in which base extension with a single cy3- or cy5-labeled nucleotide follows hybridization to a locus-specific oligomer.

Bioinformatic analyses

BeadArrays were scanned using the Illumina iScan technology, and the raw signal intensities were extracted from the *.IDAT files using the R package *methylumi*. The intensities were corrected for background fluorescence and red-green dye-bias using the methods described by Triche et al (Triche et al., 2013). The beta value was calculated as (M/(M+U)), in which M and U refer to the mean methylated and unmethylated probe signal intensities, respectively. Measurements in which the fluorescent intensity was not statistically significantly above background signal (detection p value > 0.01) were removed from the data set. We also masked all HM450 probes that have common SNPs with a minor allele frequency (MAF) greater than 1% (UCSC criteria) at the targeted CpG site, as well as probes with common SNPs (MAF>1%) within 10 bp of the targeted CpG site. HM450 probes that are within 15 bases of the CpG lying entirely within a repeat region were also masked prior to data analyses.

As a first step, missing beta-values were imputed using KNN-imputation (Troyanskaya et al., 2001) and the data underwent quantile normalization. The exported data was analyzed using the R packages Minfi, Methylumi and LIMMA available at http://bioconductor.org, as well as IMA available at www.rforge.net. After filtering out probes with a detection p-value >0.05, positioned within 15 bp of a SNP, overlapping repetitive elements, mapping to multiple locations, or located on the X and Y-chromosomes, 385,826 probes remained for downstream analysis. Beta-values of all the CpG sites were logit transformed to M-values and methylation variable positions (MVPs) were identified using the Bioconductor package *limma* (Ritchie et al., 2015). An FDR cutoff of 0.01 was applied and a minimum 0.2 difference in mean beta-value between groups was used to filter the MVPs. To identify differential methylation at the

region-level of the 11 annotated categories (e.g promoter, gene body, 3'UTR) we calculated the mean beta-values of differentially methylated CpG sites within those individual regions and filtered the data using a beta-difference of at least 0.15 between the two groups and an adjusted p-value < 0.05.

Methylation data (beta-values) and ChIP-seq data for cell lines were visualized via the ENCODE portal of the UCSC genome browser (GM12878: Gene expression omnibus (GEO) accession number GSM999376, Jurkat: GEO accession number GSM999367; ESC H3K27me3: GEO accession number GSM733748, GM12878 H3K27me3: GEO accession number GSM733758, data were produced by the Dr. Richard Myers Lab and the Dr. Devin Absher lab at the Hudson Alpha Institute for Biotechnology (Bernstein et al., 2012)) (Kent et al., 2002).

Gene expression analyses of published microarray data including ALK+ ALCL and normal T cell subsets (CD4 and CD8) (Eckerle et al., 2009) (GEO accession number GSE14879) were performed with GeneSpring (GX 11) software (Agilent). For this, we normalized the data using the robust multi-array average (RMA) summarization algorithm. We used a corrected p-value cut-off of 0.05 and adjusted for multiple testing by Benjamini – Hochberg correction. Functional annotation and GO term analysis of differentially methylated regions were performed with the Database for Annotation, Visualization and Integrated Discovery (DAVID, http://david.abcc.ncifcrf.gov) (Huang et al., 2009a; Huang et al., 2009b).

To compare our ALCL methylation data with methylation of different thymic T cell subsets we downloaded the GSE55111 dataset from the GEO data repository from a recent methylation study on different stages of T cell development (Rodriguez et al., 2015). Investigated cell types of GSE55111 covered multipotent progenitors ($CD34^+$ $CD1a^-$), thymocyte precursors

($CD34^+$ $CD1a^+$), pre-TCR thymocytes, DP $TCR\alpha ß^+$ thymocytes and $CD4^+$ and $CD8^+$ SP thymocytes. A total of 11 samples (duplicates for each stage except for $CD34^+$ $CD1a^+$) were included and compared with our ALCL and $CD3^+$ methylation profiles. Preprocessing of the 2 combined data sets comprised imputation of missing data by k-nearest neighbour (knn) algorithm and quantile normalization to ensure equal data distribution. The Qlucore Omics Explorer was used to visualize the combined data set. The different data origin introduced significant batch effects and was considered as confounding factor. This confounding factor was eliminated before analysis.

In order to identify conserved DNA sequence motifs in regions surrounding hypomethylated CpG sites, we used the MEME-ChIP tool included in the MEME suite analysis tool (Bailey et al., 2009; Machanick and Bailey, 2011). Sequences surrounding hypomethylated CpG sites by 100bp were obtained from the UCSC table browser (Karolchik et al., 2004). MEME-ChIP settings were set to default except for number of repetitions (set to “Any number of repetitions”), motif width (min=4; max=15). To compare the identified motifs with known motifs we used the TOMTOM tool within the MEME suite. For pathway and network analyses, data were analyzed through the use of QIAGEN’s Ingenuity® Pathway Analysis (IPA®, QIAGEN Redwood City,www.qiagen.com/ingenuity).

Knockdown of JunB by shRNA

ShRNA was expressed from lentiviral vectors pRS19-U6-(sh)-UbiC-TagRFP-2A-Puro containing oligos targeting *JUNB* or luciferase as control. ShRNA Oligo sequences are listed in the table below. Production of lentivirus particles for shRNA knockdown was carried out using HEK293FT cells in Opti-MEM reduced serum medium (GIBCO). ShRNAs vectors were

transfected using Lipofectamine® LTX Reagent with PLUS™ Reagent (life technologies) together with packaging plasmids psPAX2 and pMD2.G (Addgene) into HEK293FT cells according to the manufacturer's protocol. The transfection mix was removed after 24 hours and cells were further grown in DMEM (GIBCO) containing 10% FBS (fetal bovine serum) and 1% penicillin/streptomycin. Virus particles were harvested after 48 hours. Virus supernatant was added to $3x10^5$ Karpas-299 cells in RPMI supplemented with 4 µg/ml polybrene (Sigma) and cells were centrifuged for 90 min at 1000 rpm. After two days, transduced cells were selected in RPMI supplemented with 1 mg/ml puromycin for one week prior to further analysis. Then, the functionality of the shRNAs was validated by Western blot analysis. Antibodies used for Western blot were: anti-JunB (Santa Cruz, sc-8051) and anti-beta-Actin (Cell signalling, #4967). For cell cycle analysis, $3x10^5$ cells/ml of Karpas-299 transfected with shJun1, shJun2 or control were seeded in RPMI supplemented with 1 mg/ml puromycin, grown for 48 hours and stained with propidium iodide (BD Bioscience). For calculation of population doublings, $3x10^5$ cells/ml of Karpas-299 transfected with shJun1, shJun2 or control were seeded in RPMI supplemented with 1 mg/ml puromycin. After 1, 3, 4 and 5 days, cells were counted and diluted to $5x10^5$ cells/ml in fresh RPMI medium. Two independent samples of shJun1, shJun2 and control were analysed. Descriptive statistics for analysis are reported as mean ± SEM. Western blot, PI staining for cell cycle analysis and calculation of population doublings were performed as previously described (Hassler et al., 2012).

Primer and shRNA-oligo Sequences

**ms-qPCR**	
ALUfo	5'-GGTTAGGTATAGTGGTTTATATTTGTAATTTTAGTA-3',
ALUre	5'-ATTAACTAAACTAATCTTAAACTCCTAACCTCA-3';
BCL11Bfo	5'-AGGAGAAGGAGAGTTAAAGTAAAGCGAA-3',

BCL11Bre	5’-ACTACCAAACTAACAACAACGACGA-3’;
LCKfo	5’-TGATAGTAGACGGTTGTAGTTGTGC-3’,
LCKre	5’-CTACCTCCCACCTAACCTTAAACG-3’;
LEF1fo	5’-GAGTGTCGGGTATTAGGGTTTATTC-3’,
LEF1re	5’-CTTCTAACCCGCTACGAACGAT-3’;
TCF7fo	5’-GCGTATTGGAGTTTGGGTACG-3’,
TCF7re	5’-TTTCTCCTCCGACTATAAAAAACGA-3’.
**ChIP**	
IL2RGfo	5’-CCATTGACTGAGGTGGGGAAGGC-3’
IL2RGre	5’-GAGACTGGCGAGGAAGTGTGACT-3’
SAT2fo	5’-ATCGAATGGAAATGAAAGGAGTCA-3’
SAT2re	5’-GACCATTGGATGATTGCAGTCA-3’
GAPDHfo	5’-TACTAGCGGTTTTACGGGCG-3’
GAPDHre	5’-TCGAACAGGAGGAGCAGAGAGCGA-3’
HOXA9fo	5’-CGCTTAAGAAGTGTGTGTATGG-3’
HOXA9re	5’-CGTCCAGCAGAACAATAACG-3’
HOXD3fo	5’-CGCTTTGTGTGAGGCTTTCC-3’
HOXD3re	5’-CCCGTCAGGTGAAAGGAGAG-3’
PLAUfo	5’-ACGACACCTAACCCAATCCT-3’
PLAUre	5’-GGGTTTGTTTGATGGTGCTATC-3’
PDGFRBfo	5′-CAGGTCATCTGCTCCAAGTG-3′,
PDGFRBre	5′-TTGCACTGTCCTGTCTGTCC-3′;
PDGFRBnegfo	5′-GGGTATATGGCCTTGCTTCA-3′,
PDGFRBnegre	5’-GAGGAATCCCTCACCCTCTC-3’;
SERPINA1fo	5’-GAGAGACCGCTCATCCAAAG-3’
SERPINA1re	5’-GATGTGCTTCCCCACCTCTA-3’
LYNfo	5’-AAGGAGACGCGAGACGTGTA-3’
LYNre	5’-GGCTTTGAAGGCACAGAAAC-3’
TLR6fo	5’-AGAAAGGCTGGCTTCTTGTG-3’
TLR6re	5’-TTTCCCTTGGCTTGTTTCAC-3’
**shRNAs**	
shJun1	ACCGGCAGACTCGATTCATATTGAATGTTAATATTCATAGCATTCAATATGAATCGAGTCTGTTTT<br>CGAAAAAACAGACTCGATTCATATTGAATGCTATGAATATTAACATTCAATATGAATCGAGTCTGC
shJun2	ACCGGCACGACTACAAACTCTTGAAAGTTAATATTCATAGCTTTCAGGAGTTTGTAGTCGTGTTTT<br>CGAAAAAACACGACTACAAACTCCTGAAAGCTATGAATATTAACTTTCAAGAGTTTGTAGTCGTGC

Supplemental References

Bailey, T. L., Boden, M., Buske, F. A., Frith, M., Grant, C. E., Clementi, L., Ren, J., Li, W. W., and Noble, W. S. (2009). MEME SUITE: tools for motif discovery and searching. Nucleic Acids Res *37*, W202-208.

Bernstein, B. E., Birney, E., Dunham, I., Green, E. D., Gunter, C., Snyder, M., and Consortium, E. P. (2012). An integrated encyclopedia of DNA elements in the human genome. Nature *489*, 57-74.

Eckerle, S., Brune, V., Döring, C., Tiacci, E., Bohle, V., Sundström, C., Kodet, R., Paulli, M., Falini, B., Klapper, W.*, et al.* (2009). Gene expression profiling of isolated tumour cells from anaplastic large cell lymphomas: insights into its cellular origin, pathogenesis and relation to Hodgkin lymphoma. Leukemia *23*, 2129-2138.

Hassler, M. R., Klisaroska, A., Kollmann, K., Steiner, I., Bilban, M., Schiefer, A. I., Sexl, V., and Egger, G. (2012). Antineoplastic activity of the DNA methyltransferase inhibitor 5-aza-2'-deoxycytidine in anaplastic large cell lymphoma. Biochimie *94*, 2297-2307.

Huang, d. W., Sherman, B. T., and Lempicki, R. A. (2009a). Bioinformatics enrichment tools: paths toward the comprehensive functional analysis of large gene lists. Nucleic Acids Res *37*, 1-13.

Huang, d. W., Sherman, B. T., and Lempicki, R. A. (2009b). Systematic and integrative analysis of large gene lists using DAVID bioinformatics resources. Nat Protoc *4*, 44-57.

Karolchik, D., Hinrichs, A. S., Furey, T. S., Roskin, K. M., Sugnet, C. W., Haussler, D., and Kent, W. J. (2004). The UCSC Table Browser data retrieval tool. Nucleic Acids Res *32*, D493-496.

Kent, W. J., Sugnet, C. W., Furey, T. S., Roskin, K. M., Pringle, T. H., Zahler, A. M., and Haussler, D. (2002). The human genome browser at UCSC. Genome Res *12*, 996-1006.

Machanick, P., and Bailey, T. L. (2011). MEME-ChIP: motif analysis of large DNA datasets. Bioinformatics *27*, 1696-1697.

Ritchie, M. E., Phipson, B., Wu, D., Hu, Y., Law, C. W., Shi, W., and Smyth, G. K. (2015). limma powers differential expression analyses for RNA-sequencing and microarray studies. Nucleic Acids Res *43*, e47.

Rodriguez, R. M., Suarez-Alvarez, B., Mosen-Ansorena, D., Garcia-Peydro, M., Fuentes, P., Garcia-Leon, M. J., Gonzalez-Lahera, A., Macias-Camara, N., Toribio, M. L., Aransay, A. M., and Lopez-Larrea, C. (2015). Regulation of the transcriptional program by DNA methylation during human alphabeta T-cell development. Nucleic Acids Res *43*, 760-774.

Triche, T. J., Jr., Weisenberger, D. J., Van Den Berg, D., Laird, P. W., and Siegmund, K. D. (2013). Low-level processing of Illumina Infinium DNA Methylation BeadArrays. Nucleic Acids Res *41*, e90.

Troyanskaya, O., Cantor, M., Sherlock, G., Brown, P., Hastie, T., Tibshirani, R., Botstein, D., and Altman, R. B. (2001). Missing value estimation methods for DNA microarrays. Bioinformatics *17*, 520-525.
